# Platypus and echidna genomes reveal mammalian biology and evolution

**DOI:** 10.1038/s41586-020-03039-0

**Published:** 2021-01-06

**Authors:** Yang Zhou, Linda Shearwin-Whyatt, Jing Li, Zhenzhen Song, Takashi Hayakawa, David Stevens, Jane C. Fenelon, Emma Peel, Yuanyuan Cheng, Filip Pajpach, Natasha Bradley, Hikoyu Suzuki, Masato Nikaido, Joana Damas, Tasman Daish, Tahlia Perry, Zexian Zhu, Yuncong Geng, Arang Rhie, Ying Sims, Jonathan Wood, Bettina Haase, Jacquelyn Mountcastle, Olivier Fedrigo, Qiye Li, Huanming Yang, Jian Wang, Stephen D. Johnston, Adam M. Phillippy, Kerstin Howe, Erich D. Jarvis, Oliver A. Ryder, Henrik Kaessmann, Peter Donnelly, Jonas Korlach, Harris A. Lewin, Jennifer Graves, Katherine Belov, Marilyn B. Renfree, Frank Grutzner, Qi Zhou, Guojie Zhang

**Affiliations:** 1 BGI-Shenzhen, Shenzhen, China; 2 Villum Center for Biodiversity Genomics, Section for Ecology and Evolution, Department of Biology, University of Copenhagen, Copenhagen, Denmark; 3 School of Biological Sciences, The Environment Institute, The University of Adelaide, Adelaide, South Australia Australia; 4 MOE Laboratory of Biosystems Homeostasis and Protection and Zhejiang Provincial Key Laboratory for Cancer Molecular Cell Biology, Life Sciences Institute, Zhejiang University, Hangzhou, China; 5 BGI Education Center, University of Chinese Academy of Sciences, Shenzhen, China; 6 Faculty of Environmental Earth Science, Hokkaido University, Sapporo, Japan; 7 Japan Monkey Centre, Inuyama, Japan; 8 School of BioSciences, The University of Melbourne, Melbourne, Victoria Australia; 9 School of Life and Environmental Sciences, The University of Sydney, Sydney, New South Wales Australia; 10 digzyme Inc, Tokyo, Japan; 11 School of Life Science and Technology, Tokyo Institute of Technology, Tokyo, Japan; 12 The Genome Center, University of California, Davis, CA USA; 13 Department of Biomedical Engineering, Johns Hopkins University, Baltimore, MD USA; 14 Genome Informatics Section, Computational and Statistical Genomics Branch, National Human Genome Research Institute, National Institutes of Health, Bethesda, MD USA; 15 Tree of Life Programme, Wellcome Sanger Institute, Cambridge, UK; 16 The Vertebrate Genome Lab, The Rockefeller University, New York, NY USA; 17 James D. Watson Institute of Genome Sciences, Hangzhou, China; 18 University of the Chinese Academy of Sciences, Beijing, China; 19 Guangdong Provincial Academician Workstation of BGI Synthetic Genomics, BGI-Shenzhen, Shenzhen, China; 20 School of Agriculture and Food Sciences, The University of Queensland, Gatton, Queensland Australia; 21 Laboratory of Neurogenetics of Language, The Rockefeller University, New York, NY USA; 22 Howard Hughes Medical Institute, Chevy Chase, MD USA; 23 San Diego Zoo Global, Escondido, CA USA; 24 Center for Molecular Biology of Heidelberg University (ZMBH), DKFZ-ZMBH Alliance, Heidelberg, Germany; 25 Wellcome Centre for Human Genetics, University of Oxford, Oxford, UK; 26 Pacific Biosciences, Menlo Park, CA USA; 27 Department of Evolution and Ecology, College of Biological Sciences, University of California, Davis, CA USA; 28 Department of Reproduction and Population Health, School of Veterinary Medicine, University of California, Davis, CA USA; 29 Research School of Biology, Australian National University, Canberra, Australian Capital Territory Australia; 30 Institute for Applied Ecology, University of Canberra, Canberra, Australian Capital Territory Australia; 31 School of Life Sciences, La Trobe University, Melbourne, Victoria Australia; 32 Department of Neuroscience and Developmental Biology, University of Vienna, Vienna, Austria; 33 Center for Reproductive Medicine, The 2nd Affiliated Hospital, School of Medicine, Zhejiang University, Hangzhou, China; 34 State Key Laboratory of Genetic Resources and Evolution, Kunming Institute of Zoology, Chinese Academy of Sciences, Kunming, China; 35 Center for Excellence in Animal Evolution and Genetics, Chinese Academy of Sciences, Kunming, China

**Keywords:** Evolutionary genetics, Comparative genomics

## Abstract

Egg-laying mammals (monotremes) are the only extant mammalian outgroup to therians (marsupial and eutherian animals) and provide key insights into mammalian evolution^1,2^. Here we generate and analyse reference genomes of the platypus (*Ornithorhynchus anatinus*) and echidna (*Tachyglossus aculeatus*), which represent the only two extant monotreme lineages. The nearly complete platypus genome assembly has anchored almost the entire genome onto chromosomes, markedly improving the genome continuity and gene annotation. Together with our echidna sequence, the genomes of the two species allow us to detect the ancestral and lineage-specific genomic changes that shape both monotreme and mammalian evolution. We provide evidence that the monotreme sex chromosome complex originated from an ancestral chromosome ring configuration. The formation of such a unique chromosome complex may have been facilitated by the unusually extensive interactions between the multi-X and multi-Y chromosomes that are shared by the autosomal homologues in humans. Further comparative genomic analyses unravel marked differences between monotremes and therians in haptoglobin genes, lactation genes and chemosensory receptor genes for smell and taste that underlie the ecological adaptation of monotremes.

## Abstract (web summary)

New reference genomes of the two extant monotreme lineages (platypus and echidna) reveal the ancestral and lineage-specific genomic changes that shape both monotreme and mammalian evolution.

## Main

The iconic egg-laying monotremes of Australasia represent one of the three major mammalian lineages. The monotreme lineage comprises two extant families, the semi-aquatic Ornithorhynchidae (platypus) and the terrestrial Tachyglossidae (echidna). At present, the single species of platypus has a restricted distribution in Eastern Australia, whereas four echidna species (*T. aculeatus* and three *Zaglossus* spp.) are present in Australia and New Guinea (Supplementary Information). Platypuses and echidnas feature radical differences in diet (carnivorous compared with insectivorous), neurophysiology (electroreception-oriented compared with olfaction-oriented), as well as specific intraspecific conflict and defence adaptations^1^. Owing to their distinct ecological, anatomical and physiological features, monotremes are interesting mammals well-suited for the study of the evolution of ecological adaptation. Of particular interest are their sex chromosomes, which originated independently from those of therian mammals through additions of autosomes onto an ancestral XY pair, resulting in a multiple sex chromosome system that assembles as a chain during meiosis^3^.

The previous female platypus genome assembly (OANA5) provided many important insights into monotreme biology and mammalian evolution. However, only about 25% of its sequence was assigned to chromosomes^2^. The incomplete platypus assembly without Y chromosome sequences and lack of an echidna genome have limited the interpretation of the evolution of mammals and monotremes. Here we combined PacBio long-read, 10× linked-read, chromatin conformation (Hi-C) and physical map data to produce a highly accurate chromosome-scale assembly of the platypus genome. We also produced a less-continuous assembly for the short-beaked echidna, which enables us to infer the genomic changes that occurred in the ancestral monotremes and other mammals.

## Chromosome-scale monotreme genomes

**Fig. 1:**
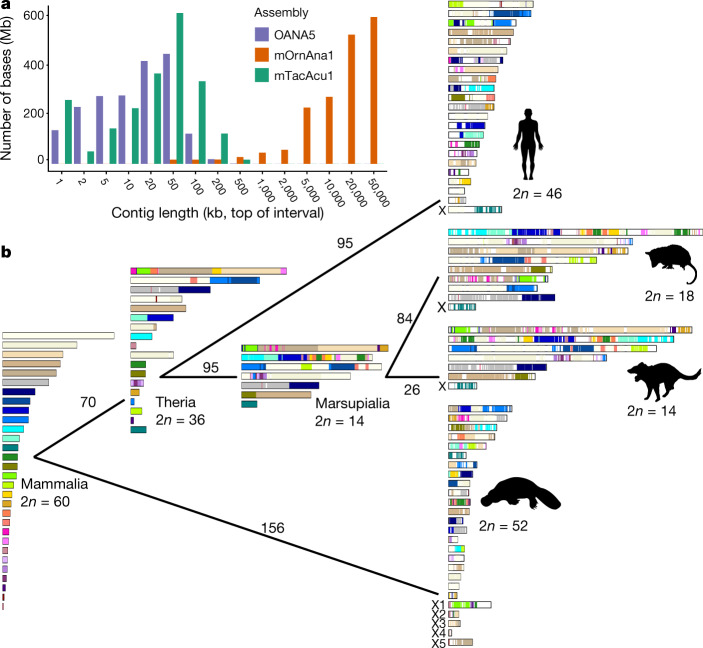
Chromosome assembly of monotreme and mammalian genome evolution. **a**, The contig length distribution among the three monotreme assemblies shows a large improvement in the sequence continuity of the platypus assembly, and at least equivalent quality of the echidna assembly. **b**, Mammalian karyotype evolution trajectory. 2*n* = 60 ancestral karyotypes were inferred for the common ancestor of mammals. Conserved blocks were colour-coded in accordance with their chromosomal source in the mammalian ancestor. Numbers of estimated rearrangements are shown for each branch. Silhouettes of the human and opossum are from https://www.flaticon.com/. Silhouettes of the platypus and Tasmanian devil are created by S. Werning and are reproduced under the Creative Commons Attribution 3.0 Unported licence (http://creativecommons.org/licenses/by/3.0/).

**Extended Data Fig. 1:**
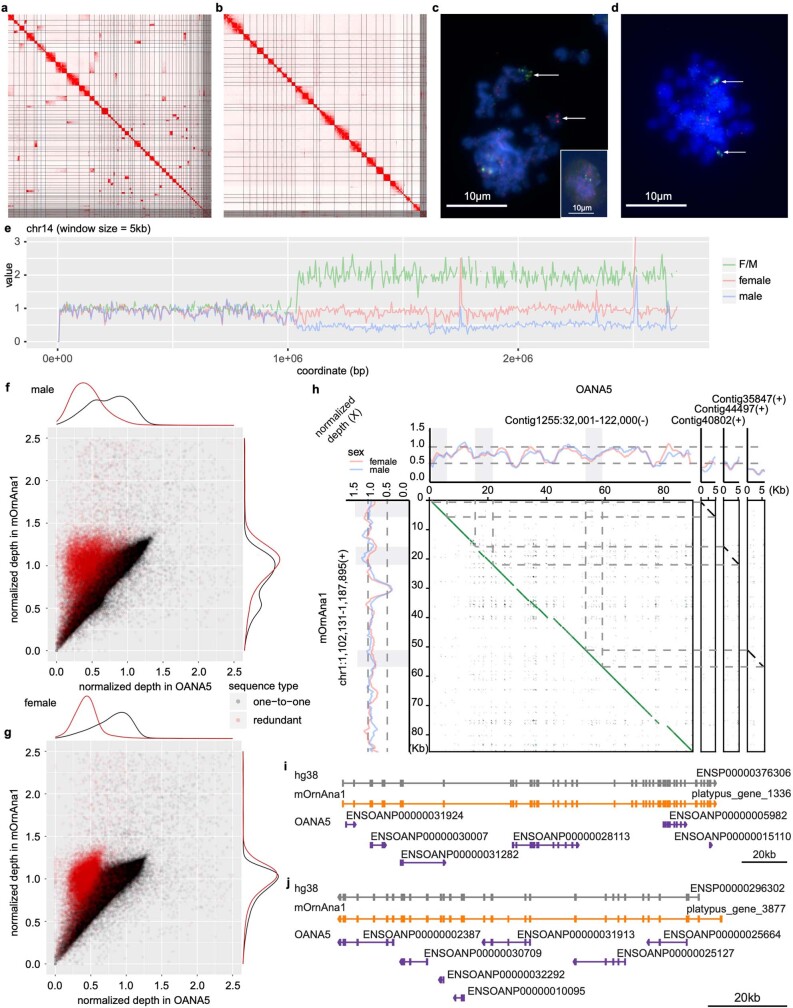
Platypus genome assembly and evaluation. **a**, **b**, Hi-C two-dimensional juicebox maps of mOrnAna1 before (**a**) and after (**b**) manual assembly curation. The grey lines depict scaffold boundaries. The off-diagonal matches between scaffolds indicate potential missed joins, whereas ‘empty’ areas within scaffold boundaries indicate misjoins. The gEVAL-supported manual assembly curation led to a notably improved arrangement with >96% of the assembly sequence inside chromosome-scale scaffolds. **c**, **d**, The Super_Scaffold_40 was misassigned to chromosome 15 in OANA5 but FISH on metaphase spreads from platypus fibroblasts map it to chromosome 13. **c**, Co-hybridization of the BAC of chromosome 15 (green, top arrow) and Super_Scaffold_40 probe (red, bottom arrow) showing an absence of co-localization (14 nuclei scored, 2 independent experiments). Inset, interphase example. **d**, Co-hybridization of the BAC of chromosome 13 (green) and Super_Scaffold_40 probe (red) showing co-localization (arrows, 40 nuclei scored, 5 independent experiments). Scale bars, 10 μm. **e**, An example of scaffold chromosome misassignment in OANA5. Female-to-male (F/M) depth ratio, normalized female depth and normalized male depth along OANA5 chromosome 14 in 5-kb non-overlapping windows. Depth ratio, normalized female depth and normalized male depth all suggest that OANA5 chromosome 14 should be an X-borne rather than autosomal sequence. **f**, **g**, Normalized depth distribution of redundant sequences and one-to-one sequences in male (**f**) and female (**g**). Redundant sequences (red) in OANA5 are probably assembly artefacts due to heterozygotes of the sequenced individual of OANA5, and are therefore featured with 0.5× normalized depth in OANA5 but 1× normalized depth in mOrnAna1 in both male and female. One-to-one sequences in OANA5 (black) have 1× normalized depth in both OANA5 and mOrnAna1 in reads that are mapped from both sexes as expected. Each dot represents one mapping region between OANA5 and mOrnAna1 by Mashmap, and the normalized depth values of each dot are calculated as the mean depth across the mapping region in OANA5 and mOrnAna1. The small peak in one-to-one sequence density plot in the male indicates candidate X-linked sequences. **h**, Example redundant sequences Contig40802, Contig44497 and Contig35847 in OANA5 that could be interpreted as false duplications. Dot plot is generated between the target region of mOrnAna1 chromosome 1 and OANA5 Contig1255, Contig40802, Contig44497 and Contig35847 by FlexiDot. Candidate redundant sequences are those mapped to the same region in mOrnAna1 chromosome 1, highlighted by dashed lines in the dot plot and grey in the normalized depth plot. Normalized male and female read depths along each sequence are calculated in 500-bp windows, and plotted along each sequence. Although the normalized depth is always around 1 in the region of mOrnAna1 chromosome 1, normalized depth drops half in Contig40802, Contig44497, Contig35847 and the aligned regions in Contig1255, indicating that Contig40802, Contig44497 and Contig35847 are probably redundant sequences in OANA5. **i**, **j**, Examples of gene annotation artefacts in OANA5: *CIT* (**i**) and *PBRM1* (**j**) have been fragmented into multiple small artificial genes in OANA5 (purple) but have now been fully recovered in mOrnAna1 (orange). Orthologous human genes (grey) are also shown to indicate that the mOrnAna1 rather than OANA5 annotation has a similar gene structure to that of the human genes.

Our new male platypus genome assembly (mOrnAna1) shows a 1,390-fold improvement for the contig N50 and a 49-fold improvement for the scaffold N50 compared with the previous Sanger-based assembly (OANA5) (Fig. 1a). We performed extensive error correction and manual curation to polish and anchor the assembly at the chromosome scale (Extended Data Fig. 1a, b). Ambiguous chromosome assignments were resolved with fluorescence in situ hybridization (FISH) experiments (Extended Data Fig. 1c, d). We also produced a male echidna genome (mTacAcu1) from a variety of short- and long-insert-size libraries, and further scaffolded it using the same methods as in platypus. The resulting mTacAc1 sequence shows better sequence continuity than OANA5, with a scaffold N50 size of 32.51 Mb (Supplementary Table 2).

To study the origin and evolution of monotreme sex chromosomes, we greatly improved the assembly of the platypus sex chromosomes. We anchored 172 Mb (92% compared to 22% in OANA5) X-borne sequences to chromosomes (Supplementary Tables 4, 6). This includes one 1.6-Mb segment that was previously misassigned to chromosome 14 (Extended Data Fig. 1e). We determined all of the pseudoautosomal regions (PARs) except for X4, on the basis of the different read coverage between sexes and representation of FISH markers (Supplementary Table 3). We also mapped 92% of the platypus Y-borne sequences to the five Y chromosomes using PacBio reads produced using Y-borne bacterial artificial chromosome (BAC) clones^4^ (Supplementary Tables 5, 6). Owing to a lack of echidna linkage markers, we used the platypus X chromosomes as a reference to anchor a similar length (177 Mb, 96%) of X chromosomes and identified 8.6 Mb Y-borne sequences in echidna.

In the final curated platypus genome (mOrnAna1) 98% of the sequence was assigned to the 21 autosomes, 5 X and 5 Y chromosomes (Supplementary Table 7), with putative telomeres and centromeres annotated for half of the chromosomes (Supplementary Table 8). mOrnAna1 fills around 90% of the gaps in OANA5 (Supplementary Table 9), recovering 161 Mb of previously missed genomic sequences, most of which are long interspersed nuclear elements (LINE)/L2 and short interspersed nuclear elements (SINE)/MIR (Supplementary Tables 10, 11). We also removed 68 Mb of redundant sequences in OANA5 (Extended Data Fig. 1f–h). The repeat elements comprising about half of the monotreme genomes are dominated by LINE/L2 elements that are more similar to reptile genomes than therian mammals (which comprise mostly LINE/L1)^5^ (Supplementary Table 12). The highly continuous assembly also substantially improves gene annotation. We identified 20,742 and 22,029 protein-coding genes in mOrnAna1 and mTacAcu1, respectively (Supplementary Table 13). Specifically, 19,576 coding exons from 8,303 platypus genes were recovered from the gapped regions of OANA5. Among them, 454 genes were completely missed in OANA5, and 3,961 fragmented genes in OANA5 now have complete open-reading frames. We corrected 2,395 genes that were previously split or misannotated in OANA5 (Extended Data Fig. 1i, j).

## Insights into mammalian genome evolution

**Extended Data Fig. 2:**
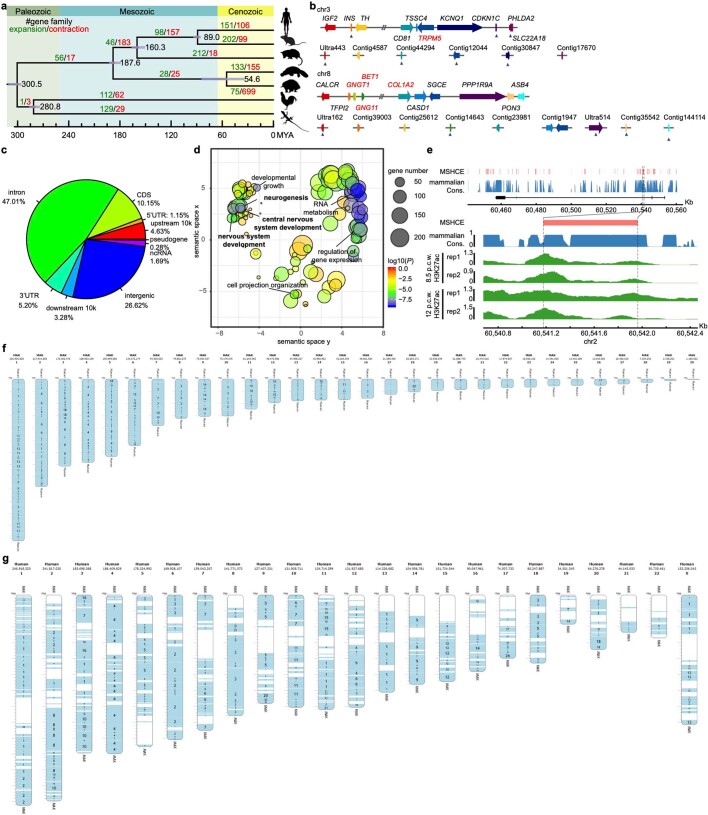
Mammalian genome evolution. **a**, Phylogenetic tree constructed using fourfold degenerate sites from 7,946 one-to-one orthologues among seven representative species (human, mouse, opossum, platypus, echidna, chicken and green lizard). The fossil time calibration of the nodes marked by circles were obtained from a previously published study^67^. The numbers of gene families that have undergone significant (Viterbi *P* < 0.05) lineage-specific expansions (green) and contractions (red) are marked on each branch. Exact *P* values are available in Supplementary Table 29. No multiple-testing correction was applied. **b**, Examples of some imprinting gene clusters improved in mOrnAna1 compared to OANA5. The first line of each synteny plot represents mOrnAna1 and the second line represents OANA5. Names of genes that have been found to be imprinted in human and mouse are highlighted in black, and non-imprinting genes in red. Fragmented genes with alignment rate lower than 70% are marked by triangles. The double slash represents the intermediate region longer than 100 kb. **c**, Distribution of MSHCEs on genomic elements. **d**, Enriched GO terms in the top-300 MSHCE-associated genes. *P* values of enrichment are calculated using a *χ*^2^ test, and FDRs are computed to adjust for multiple testing. GO terms are clustered based on semantic similarity. GO terms related to nervous system development are highlighted in bold. **e**, A case of one MSHCE in *BCL11A* that overlaps with the enhancer signals inferred from H3K27ac ChIP-seq experiments at 8.5 and 12 weeks after conception (p.c.w.). **f**, Evolution highway comparative chromosome browser visualization of reconstructed MACs at a 500-kb resolution. Blocks overlaid on each MAC represent human syntenic fragments. Numbers within blocks indicate the homologous human chromosome. **g**, Evolution highway comparative chromosome browser visualization of the human genome at a 500-kb resolution, with each block overlaid on each human chromosome representing putative chromosome fragments of the ancestral mammalian genome. Numbers within blocks depict the ancestral mammal chromosome numbers. Silhouettes of the human and opossum are from https://www.flaticon.com/. The silhouette of the platypus is created by S. Werning and is reproduced under a Creative Commons Attribution 3.0 Unported licence (http://creativecommons.org/licenses/by/3.0/).

Our phylogenomic reconstruction shows that monotremes diverged from therians around 187 million years ago, and the two monotremes diverged around 55 million years ago (Extended Data Fig. 2a). This estimate provides a date for the monotreme–therian split that is earlier than previous estimates (about 21 million years ago)^2^, but agrees with recent analyses of few genes and fossil evidence^6^. We also inferred that monotremes had similar genome substitution rates (approximately 2.6 × 10^−3^ substitutions per site per million years) compared with other mammals (Supplementary Table 15). About 14 Mb of mammalian specific highly conserved elements were identified by comparison among vertebrates (Methods): around 90% of elements were located in non-coding regions (Extended Data Fig. 2c), and are associated with genes that are enriched in processes such as brain development (Extended Data Fig. 2d, e, Supplementary Results and Supplementary Tables 18–20).

Next we used chromosome information from human, opossum, Tasmanian devil, platypus, chicken and common wall lizard genomes to reconstruct the mammalian ancestral karyotype (Methods). This analysis reveals 30 mammalian ancestral chromosomes (MACs) (2*n* = 60) at a resolution of 500 kb, covering around 66% of the human genome and approximately 67% of the platypus genome (Fig. 1b and Supplementary Tables 24–26). Of these, 25 MACs were maintained without breaks in a single chromosome of the therian ancestor, and 17 of them have fused with other MACs in therians. Sixteen MACs were still maintained in a single human chromosome, but only MAC28 had not undergone any intrachromosomal rearrangements during therian evolution (Extended Data Fig. 2f, g). We detected at least 918 chromosome breakage events, and confirmed that the X chromosome in humans was derived from the fusion of an original therian X chromosome with an autosomal region after the divergence from marsupials^7^ (Fig. 1b and Extended Data Fig. 2f, g). The five X chromosomes in platypus were derived from different MACs by multiple fusion and translocation events.

**Extended Data Fig. 3:**
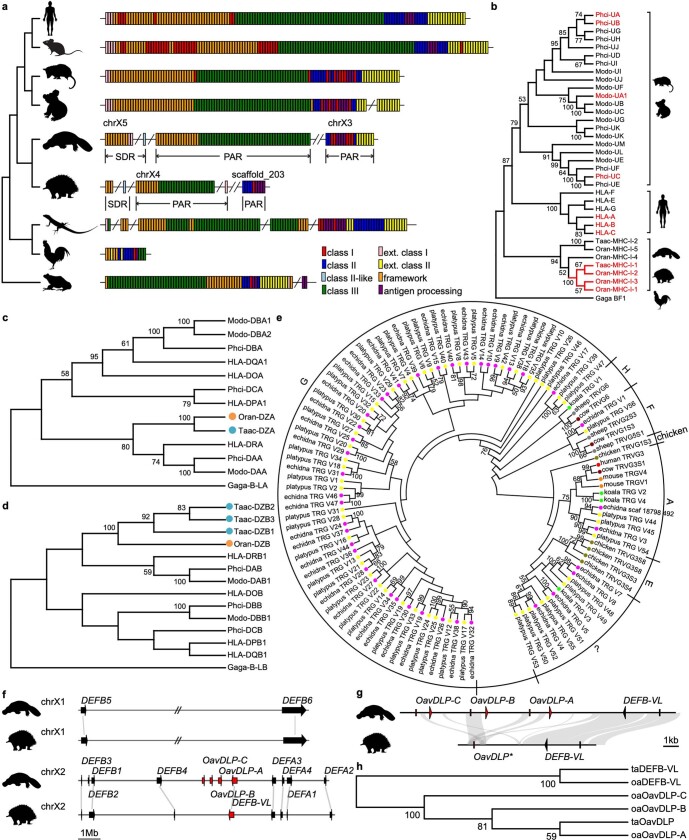
Evolution of immune gene family in monotremes. **a**, MHC genes in platypus and echidna are located on two different chromosomes, but the classical class I and II genes involved in antigen presentation are located within a single cluster in each genome. **b**, Phylogenetic relationship of class I genes in representative mammals and chicken. Classical class I genes (red) in monotremes exhibit high similarity, which is rarely observed in other species. Only bootstrap values with >50% support are shown. **c**, **d**, Phylogenetic relationship of MHC class II alpha (**c**) and beta (**d**) genes. Genes with prefix ‘HLA’, ‘Modo’, ‘Phci’, ‘Oran’, ‘Taac’ and ‘Gaga’ indicate genes in human, opossum, koala, platypus, echidna and chicken, respectively. Only bootstrap values with >50% support are shown. **e**, Phylogenetic relationship among putative functional Vγ sequences from platypus (yellow), echidna (purple), koala (green), mouse (orange), human (red), sheep (grey), cow (dark red) and chicken (dark yellow). Groups according to a previous study^90^ are displayed around the outside of the tree, with the putative marsupial–monotreme-specific group denoted by a ‘?’. Only bootstrap values with greater than 50% support are shown. **f**, Synteny conservation of beta-defensin genes in monotremes and loss of functional venom defensins in echidna. Venom defensins (OavDLP genes) and venom-like defensin (DEFB-VL genes) are shown in red. Only putative functional defensins are shown. **g**, Putative OavDLP loss in echidna. OavDLP genes and *DEFB-VL* each contain two exons (indicated by a box and triangle) in platypus. Both exons of platypus *DEFB-VL* can be mapped to echidna chromosome X2. A single platypus OavDLP exon can be mapped to echidna chromosome X2 while the second exons cannot. Grey links indicate platypus–echidna LASTZ alignment. **h**, Phylogenetic relationship of *DEFB-VL* and OavDLP genes suggested that ancestral monotremes had all three OavDLP genes but that echidna has lost the two of them (*OavDLP-B* and *OavDLP-C*). Branch length is not shown. ta, echidna; oa, platypus. Silhouettes of the human, opossum, koala and frog are from https://www.flaticon.com/. The silhouette of the platypus is created by S. Werning and is reproduced under a Creative Commons Attribution 3.0 Unported licence (http://creativecommons.org/licenses/by/3.0/).

We found that gene families associated with the immune response and hair growth were expanded considerably in the mammalian ancestor, perhaps contributing to the evolution of immune adaptation and fur, respectively, in mammals (Supplementary Table 30). We further manually annotated major histocompatibility complex (MHC) genes and other immune genes (Supplementary Results). As in nonmammalian vertebrates, the monotreme MHC class Ia genes colocalize with antigen-processing genes and MHC class II genes (Extended Data Fig. 3a and Supplementary Table 31). The defensin genes gave rise to unique defensin-like peptides (OavDLP genes) in platypus venom^8^. By contrast, echidna has only one single *OavDLP* pseudogene (Extended Data Fig. 3f–h), suggesting the loss of the key venom gene family in this species.

## Monotreme sex chromosome evolution

**Fig. 2:**
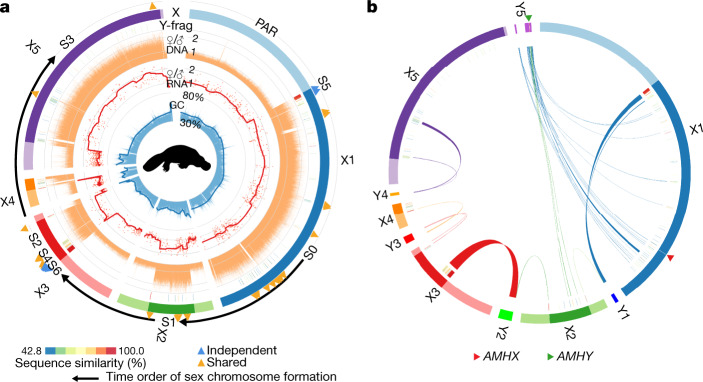
Origin and evolution of the sex chromosomes of the platypus. **a**, Genomic composition of the platypus sex chromosomes. From the outer to inner rings: the X chromosomes with the PARs (light colours) and SDRs (dark colours) labelled; the assembled Y chromosome fragments within SDRs showing the colour-scaled sequence divergence levels with the homologous X chromosomes; female-to-male (F/M) ratios of short sequencing-read coverage in non-overlapping 5-kb windows; F/M expression ratios (each red dot is one gene) of the adult kidney and the smoothed expression trend; and GC content in non-overlapping 2-kb windows. In addition, we labelled the positions on the X chromosome ring of the gametologue pairs that have suppressed recombination before the divergence of monotremes (‘shared’, orange triangles) or after the divergence (‘independent’, blue triangles). **b**, Homology between X and Y chromosomes of platypus. In particular, most of Y5 shows homology with X1 and X2, which suggests an ancestral ring conformation of the platypus sex chromosomes. We also labelled the position of the putative sex-determining gene *AMH*. The platypus silhouette is created by S. Werning and is reproduced under the Creative Commons Attribution 3.0 Unported licence (http://creativecommons.org/licenses/by/3.0/).

**Extended Data Fig. 4:**
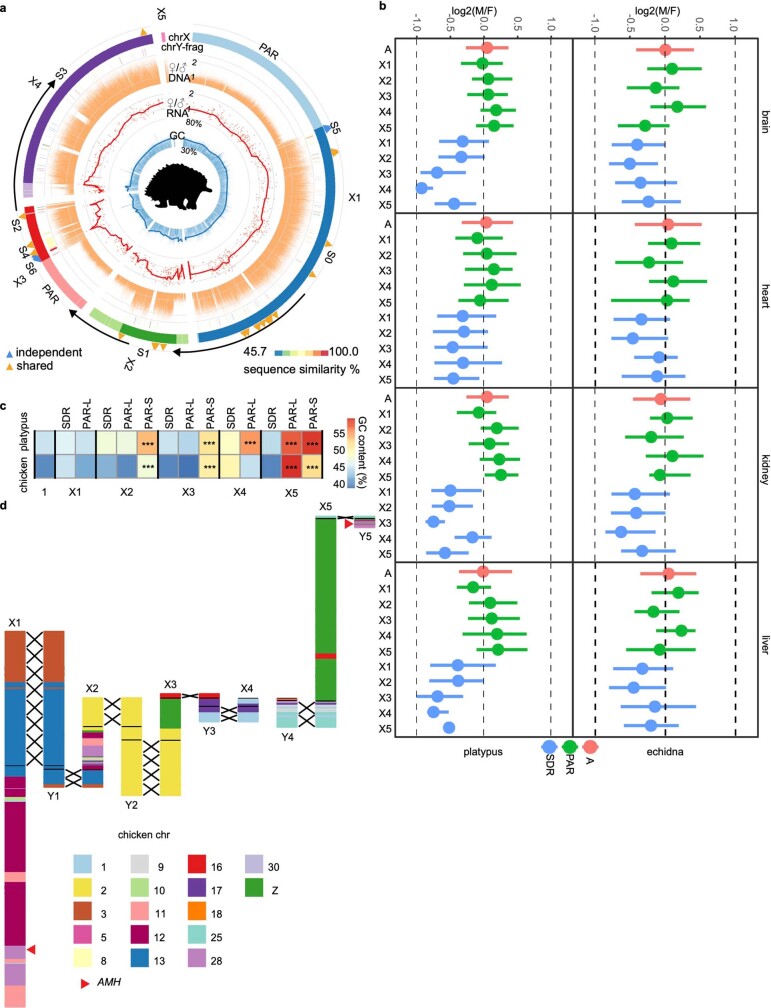
Genomic composition of monotreme sex chromosomes. **a**, Composition of the echidna sex chromosomes. The circos plot (from outer to inner rings) shows: X chromosomes with PARs shown as light colours and SDRs as dark colours; assembled Y chromosome fragments showing the colour-scaled sequence similarity levels with homologous X chromosomes; normalized F/M ratios of Illumina DNA-sequencing depth in non-overlapping 5-kb windows; F/M expression ratios (each red dot is one gene) of adult kidney and smoothed expression ratio trend; and GC content in non-overlapping 2-kb windows. In addition, Y-linked fragments with a similar level of sequence divergence from the X chromosome indicate a pattern of evolutionary strata. As expected, F/M DNA depth ratio is centred at 1 at PARs, but is around 2 at SDRs. Some PARs show significantly higher GC content than the regions that suppressed recombination between X and Y. **b**, Partial dosage compensation in monotremes. The four point range plots show log_2_-transformed values of the male-to-female (M/F) expression ratio in the brain, kidney, heart and liver of platypus and echidna. As expected, log_2_-transformed values of the M/F expression ratio is close to 0 for genes on autosomes (A) and PARs, whereas for genes on SDRs, the expression is female-biased in all tissues, which suggests that monotremes have partial dosage compensation. Whiskers indicate the 25– 75th percentiles and circles are the median value. **c**, Some PARs show significantly higher GC content than SDRs. For platypus, some PARs (X2-PAR-S, X3-PAR-S, X4-PAR-L, X5-PAR-S and X5-PAR-L (where -S is the shorter PAR of the chromosome and -L the longer PAR)) show significantly (*P* < 0.01) higher GC content (1-kb non-overlapping windows) than the SDRs of the same chromosome, which are labelled as asterisks in the heat map. We also checked their orthologous sequences in chicken, as a proxy for the ancestral status before the chromosome became a sex chromosome, and found similarly higher GC content in the orthologous region of PARs than those of SDRs in chicken. ***P < 0.01 (all *P* < 2.2 × 10^−16^), one-sided Wilcoxon rank-sum test. **d**, Atlas of orthologous chicken fragments along each platypus sex chromosome. The PARs between the platypus X and Y chromosomes are indicated by crosses. We also labelled the position of the putative sex-determining gene *AMH*.

To elucidate the detailed genomic composition of the monotreme sex chromosomes, we compared regions that share sequences between the sex chromosomes—that is, the PARs—with regions that have become sexually differentiated (SDRs). PAR boundaries show a sharp shift in the female-to-male sequencing coverage ratio as expected (Fig. 2a and Extended Data Fig. 4a). Both monotremes showed generally nonbiased gene expression levels between sexes within PARs, but pronounced female-biased expression within SDRs, indicating the absence of complete chromosome-wide dosage compensation in monotremes as previously suggested^9^ (Extended Data Fig. 4b).

The short PARs of platypus chromosomes X2–X5 have a significantly higher GC content (one-sided Wilcoxon rank-sum test, *P* < 0.01) than the SDRs or the longer PARs (Extended Data Fig. 4c), which probably reflects strong GC-biased gene conversion that is caused by a high recombination rate^10^. This is similar to the pattern of the short GC-rich human PAR, the recombination rate of which is 17-fold higher than the genome-wide average^11^. Notably, chicken orthologous sequences of these monotreme PARs are all located on the microchromosomes, which also have a high GC content^12^ (one-sided Wilcoxon rank-sum test, *P* < 0.01) (Extended Data Fig. 4c, d). This highly conserved recombination landscape might be partially selected in monotremes for maintaining the sequence polymorphism and balanced dosage of MHC genes, which reside in the PARs of the chromosome X3–Y3 and Y4–X5 pairs in platypus^13^ (Extended Data Fig. 3a). The regional selection for high recombination may also counteract further expansion of SDRs on these sex chromosomes.

**Extended Data Fig. 5:**
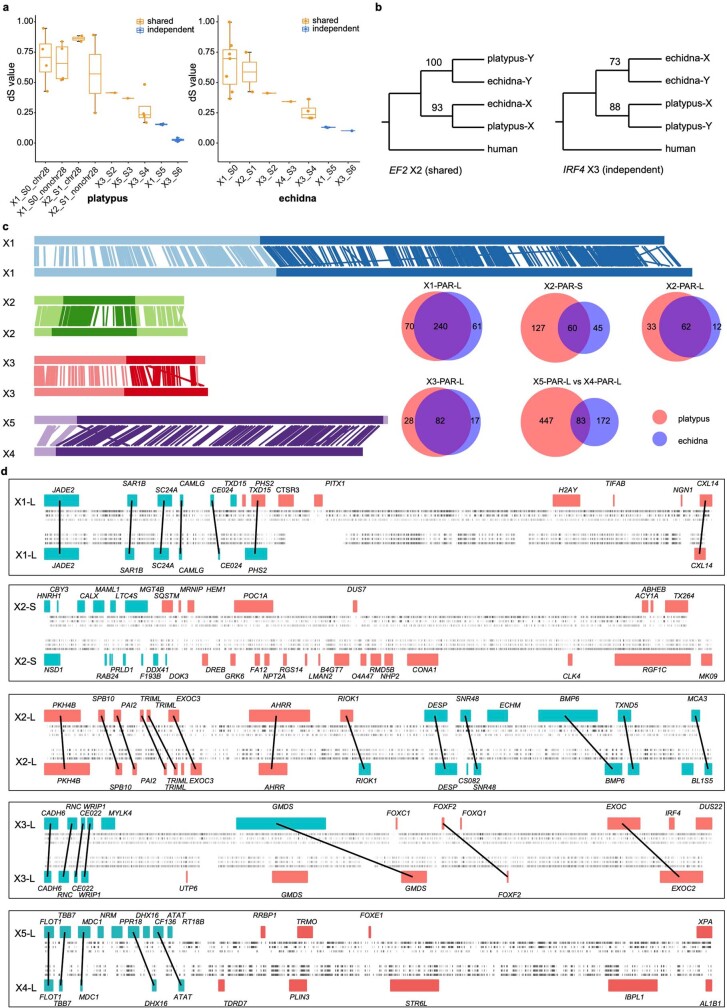
Evolution of PARs after the platypus and echidna divergence. **a**, The distribution of pairwise d*S* values of platypus and echidna sex chromosomes. In both platypus and echidna, gametologue pairs in the X1 S0 region (Fig. 2), which is largely homologous to chicken chromosome 28, have a higher d*S* value than those of any other sex-linked regions. This suggests that X1 S0 is the oldest evolutionary stratum. Therefore, we also show platypus genes of X2 with an orthologue on chicken chromosome 28 separately from others (X1_S0_chr28). Following the order of d*S* values of different chromosome regions, we inferred the time order of formation of evolutionary strata, called S0–S6. For platypus, *n* = 5, 5, 2, 2, 1, 1, 4, 2 and 6 XY gametologue pairs are plotted, from left to right. For echidna, *n* = 7, 2, 1, 1, 4, 2 and 1 XY gametologue pairs are plotted, from left to right. Box plots show median, quartiles (boxes) and range (whiskers). **b**, Phylogenetic tree examples of gametologues that evolved in the common ancestor of monotremes (*EF2* in X2) and independently in two monotreme species (*IRF4* in X3). **c**, Alignments of platypus and echidna X chromosomes (PAR, light colours; SDR, dark colours; the top chromosomes are from platypus and the bottom chromosomes are from echidna) were used to infer X2-PAR-S and X5-PAR-L of platypus evolved independently from echidna after their divergence, given their different lengths. This is supported by the Venn diagrams of PAR genes between platypus and echidna, in which most genes are not shared within independently evolved PARs. **d**, Alignments of PAR–SDR boundaries between platypus and echidna. Alignments of genes (±1 Mb around the boundaries) support independent evolution of X2-PAR-S and X5-PAR-L in platypus and echidna, as most of their genes are not homologous at the PAR–SDR boundaries (blue, PAR genes; red, SDR genes; platypus, top chromosome, echidna, bottom chromosome). We used lines to connect the genes of the two species, whenever they are orthologous to each other. For each X chromosome, we also labelled their repeat information. Six repeat tracks between each X pair are shown, from top to bottom: the overall repeat content of platypus; LINE/L2 elements of platypus; SINE/MIR elements of platypus; SINE/MIR elements of echidna; LINE/L2 elements of echidna and overall repeat content of echidna. We did not find obvious repeat enrichment at PAR–SDR boundaries, as shown previously in cow^91^.

Sex chromosomes of both eutherians and birds formed through stepwise suppression of recombination, resulting in a pattern of pairwise sequence divergence between SDRs termed ‘evolutionary strata’^14,15^. We identified at least seven strata in monotremes, named S0 to S6 from the oldest to the youngest strata (Fig. 2a and Extended Data Fig. 4a), by ranking their levels of pairwise synonymous sequence divergence between the X–Y gametologues and the phylogeny (Extended Data Fig. 5a, b). All but the most recent strata (S5 and S6) are shared by platypus and echidna. However, the PARs that border S5 and S6, as well as the shorter PARs of chromosomes X2 and X5 (Extended Data Fig. 5c, d), formed independently after their divergence. Overall, the distribution of evolutionary strata suggested a time order of incorporating different ancestral autosomes into the sex chromosome chain: it started from the S0 region of X1 containing a sex-determining gene (see below), followed by X2, X3 and X5. X4 and individual regions of X3 and X1 underwent suppression of recombination after the monotreme divergence.

**Extended Data Fig. 6:**
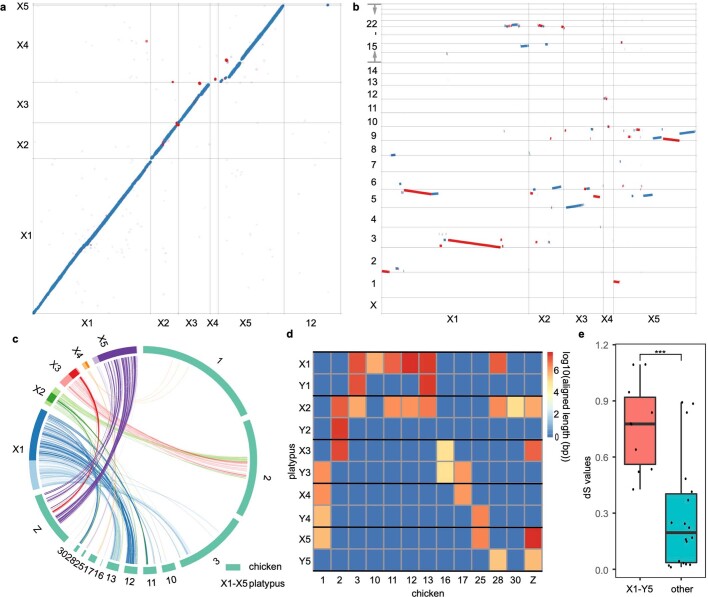
Sex chromosome evolution in monotremes. **a**, Mummerplot showing homology between platypus (*x* axis) and echidna (*y* axis) X chromosomes. Blue lines: forward alignment; red lines: reverse alignment. For echidna, X1, X2 and X3 are homologous to platypus X1, X2 and X3, respectively. Echidna X4 is homologous to platypus X5. And for echidna X5, it is not homologous to any platypus sex chromosome, and instead it is homologous to platypus chromosome 12. **b**, Homology between platypus X chromosomes (*x* axis) and human chromosomes (*y* axis). **c**, Homologous relationships between platypus sex chromosomes and chicken. **d**, Alignment between platypus and chicken showing the alternating pairing pattern of the platypus sex chromosome chain. **e**, X/Y pairwise d*S* comparison between gametologues on X1–Y5 pair (*n* = 18) and other sex chromosome pairs (*n* = 10). Box plots show median, quartiles (boxes) and range (whiskers). ***P < 0.001 (*P* = 0.0002954), one-sided Wilcoxon rank-sum test.

Despite episodes of independent evolution, most sex chromosome regions of the platypus and echidna are homologous (Extended Data Fig. 6a), suggesting that the complex formed in the monotreme ancestor^16^. To reconstruct its origin, we projected the platypus sex chromosomes onto their chicken homologues (Supplementary Table 39). This refined homology map (Extended Data Fig. 4d) suggests that both fusions and reciprocal translocations among the ancestral micro- and macrochromosomal fragments gave rise to the monotreme sex chromosome complex. The platypus X chromosomes contain homologous sequences of the entire or partial chicken microchromosomes 11, 16, 17, 25 and 28. These microchromosomes also have orthologues in the spotted gar^17^, suggesting that they were ancestral vertebrate microchromosomes, and fused in the ancestral monotreme or mammalian chromosomes. Evidence of reciprocal translocations came from the observation that parts of every two neighbouring sex chromosomes are homologous to two adjacent regions of the same chicken chromosome (Extended Data Fig. 6c, d). For example, platypus chromosomes X1 and X2 are both homologous to parts of chicken microchromosome 12 and chromosome 13, whereas X2 and X3 are both homologous to chicken chromosome 2.

Notably, X1 at one end of the meiotic chain and Y5 at the other share this alternately overlapping relationship, and both are homologous to chicken microchromosome 28. Indeed, most of the genes on Y5 are not found on its pairing partner X5, but on X1 (Fig. 2b and Supplementary Table 40). Chromosomes X1 and Y5 do not pair at meiosis, but this homology suggests that the origin of the extant monotreme sex chromosome complex involved the opening of the ancestral chromosomal ‘ring’ as degeneration proceeded^18^. A conserved vertebrate sex-determining gene, the anti-Mullerian hormone, is located on chromosome Y5 (*AMHY*) and S0 of chromosome X1 (*AMHX*)^14^ (Fig. 2b). The ancestral X1–Y5 pairing region that encompasses *AMH* could, therefore, be the site at which homologous recombination was first suppressed. The degeneration of chromosome Y5 then caused the loss of homology with X1 and led to the break of the chromosome ring. Indeed, synonymous substitution rates (d*S*) between the retained X1–Y5 gametologue pairs are significantly higher (one-sided Wilcoxon ranked-sum test, *P* < 0.01) than those of any other sex chromosome pairs (Extended Data Fig. 6e). A chromosome ring configuration has been reported in plants^19^, but not in any animal species. Alternatively, the ancestral ring structure might have evolved after the emergence of the proto-X1–Y5 pair by translocations that involve other autosomes, so that sexually antagonistic alleles could be linked to the sex-determining genes^20^.

## Interactions between sex chromosomes

**Fig. 3:**
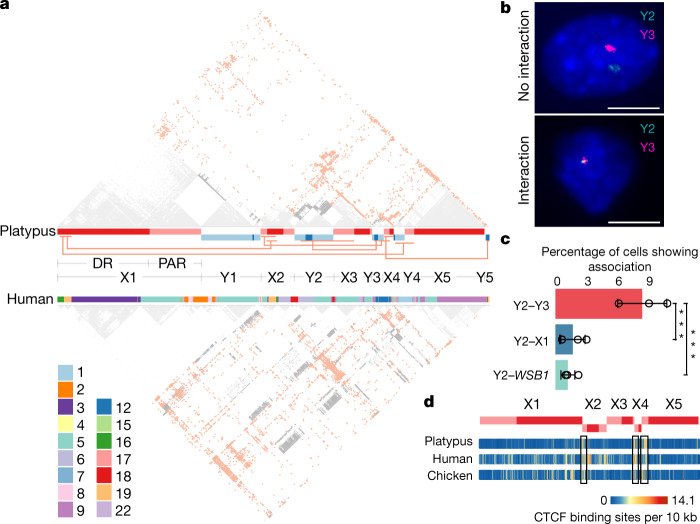
Interactions between the platypus sex chromosomes. **a**, Interchromosomal interactions among the platypus sex chromosomes detected by Hi-C data of liver tissue in platypus (top) and human (bottom). The bars between the Hi-C panels show the platypus sex chromosomes and their orthologues in the human genome. Grey, intrachromosomal interactions; red, interchromosomal interactions. Red lines link the regions with significantly high interchromosomal interactions. The interchromosomal interactions seem to be conserved in mammals, as indicated by the homologous chromosomal fragments of the human and platypus sex chromosomes and their Hi-C contact patterns. **b**, FISH with BAC probes to detect sex chromosomes Y2, Y3 or X1 and autosome chromosome17 (*WSB1*) in interphase platypus fibroblasts. Examples show no interaction between chromosomes Y2 and Y3 (top, *n* = 593, 3 independent experiments) and interaction (bottom, *n* = 56, 3 independent experiments). Scale bars, 10 μm. **c**, The significantly higher frequency of interaction between Y2 and Y3 than that between Y2 and X1, and between Y2 and *WSB1* (chromosome 17). *n* = 185, 206, 258 cells for the three independent replicate experiments of Y2–Y3, *n* = 258, 250, 205 cells for the three independent replicate experiments of Y2–X1, *n* = 298, 262, 220 cells for the three independent replicate experiments of Y2–*WSB1*. Data are mean ± s.d. ***P < 0.001 (Y2–Y3 versus Y2–X1, *P* = 0.0004675; Y2–Y3 versus Y2–*WSB1*, *P* = 6.376 × 10^−5^), one-sided Fisher’s exact test. **d**, Putative CTCF-binding-site density plot showing its enrichment among homologous regions in the platypus, human and chicken genomes.

**Extended Data Fig. 7:**
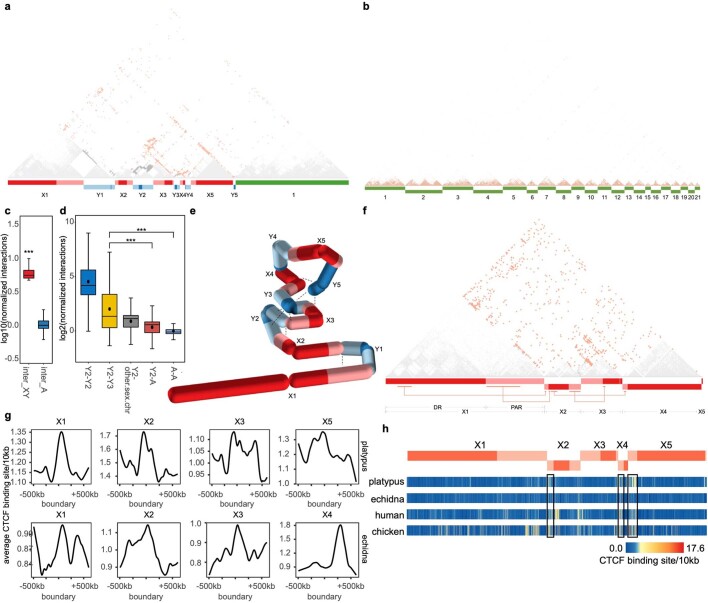
Chromatin conformation of monotreme sex chromosomes. **a**, Hi-C interactions between platypus sex chromosomes, with chromosome 1 shown as control. **a**, **b**, There are unexpected interchromosomal interactions (shown in red) between platypus sex chromosomes detected by Hi-C data (**a**), whereas most interactions are within the same chromosomes (shown in red in **b**) for the other chromosomes (**b**). **c**, The Hi-C interchromosomal interactions among platypus sex chromosome (inter_XY, *n* = 2,711 100-kb windows) is significantly higher than that among autosomes (inter_A, *n* = 14,342,930 100-kb window). Box plots show median, quartiles (boxes) and range (whiskers). ***P < 0.0001 (*P* < 2.2 × 10^−16^), one-sided Wilcoxon rank-sum test. **d**, The interaction strength is higher between Y2 and Y3 than the interaction strengths between Y2 and other chromosomes. *n* = 1,002, 228, 5,025, 67,313 and 6,904,867 100-kb windows are shown in Y2-Y2, Y2-Y3, Y2-other.sex.chr, Y2-A and A-A, respectively. Box plots show median, quartiles (boxes) and range (whiskers). ***P < 0.0001 (*P* < 2.2 × 10^−16^), one-sided Wilcoxon rank-sum test. **e**, Inferred three-dimensional structure of the platypus sex chromosome system. X chromosomes are shown in red and Y chromosomes in blue, with PARs in light colour. Interchromosomal interactions inferred from Hi-C are shown by dashed lines. **f**, Hi-C interactions reveal unexpected interchromosomal interactions between the echidna sex chromosomes. **g**, Putative CTCF-binding sites are enriched at TAD boundaries in platypus and echidna sex chromosomes. For each X chromosome of platypus, we calculated their putative CTCF-binding-site density per 10 kb and plotted them along the ±500 kb of TAD boundaries. Platypus X4 and echidna X5 are not shown because less than 10 TAD boundaries are detected. **h**, Putative CTCF-binding-site density plot showing its enrichment among the homologous regions of platypus, echidna, human and chicken.

The platypus sex chromosomes exhibit an unusual association with each other compared to autosomes during and after meiosis^21^. As little is known about their spatial organization in platypus somatic cells, we investigated this using Hi-C data (male liver) and chromosomal FISH with sex-chromosome-specific and autosomal BAC probes (male fibroblasts). Notably, Hi-C data showed that chromosomes Y2 and Y3 undergo frequent interchromosomal interactions, whereas autosomes confine their interactions mostly within chromosomes (Fig. 3a and Extended Data Fig. 7a–d). FISH showed that chromosomes Y2 and Y3 signals overlapped more frequently (5.2- and 7.6-fold) than signals between chromosomes Y2 and X1 or Y2 and an autosome (chromosome 17) (*P* = 8.67 × 10^−4^ and 8.57 × 10^−5^, respectively) (Fig. 3b, c and Supplementary Table 41). These interactions allow us to predict a zigzag three-dimensional conformation of the sex chromosomes at interphase (Extended Data Fig. 7e). A similar pattern was also present in echidna (Extended Data Fig. 7f). Notably, the high interaction frequency is conserved in human orthologous autosomal regions (Fig. 3a), suggesting functional importance unrelated to the evolution or function of sex chromosomes.

We further examined the distribution of putative binding sites of the CTCF protein, which is usually enriched at the boundaries of topologically associated domains (TADs) and mediates both intra- and interchromosomal interactions^22^. This revealed considerable enrichment of putative CTCF-binding sites at the TAD boundaries of the platypus genome (Extended Data Fig. 7g), which are more enriched along the interacting sex chromosomes X2 and X4, as well as along their orthologous regions in human and chicken (Fig. 3d and Extended Data Fig. 7h). These results suggest that an ancestral interaction landscape facilitated by local enrichment of CTCF-binding sites could have promoted the reciprocal translocations between spatially adjacent autosomal fragments that gave rise to the sex chromosome complex in the monotremes.

## Eco-evolutionary adaptation of diet

**Extended Data Fig. 8:**
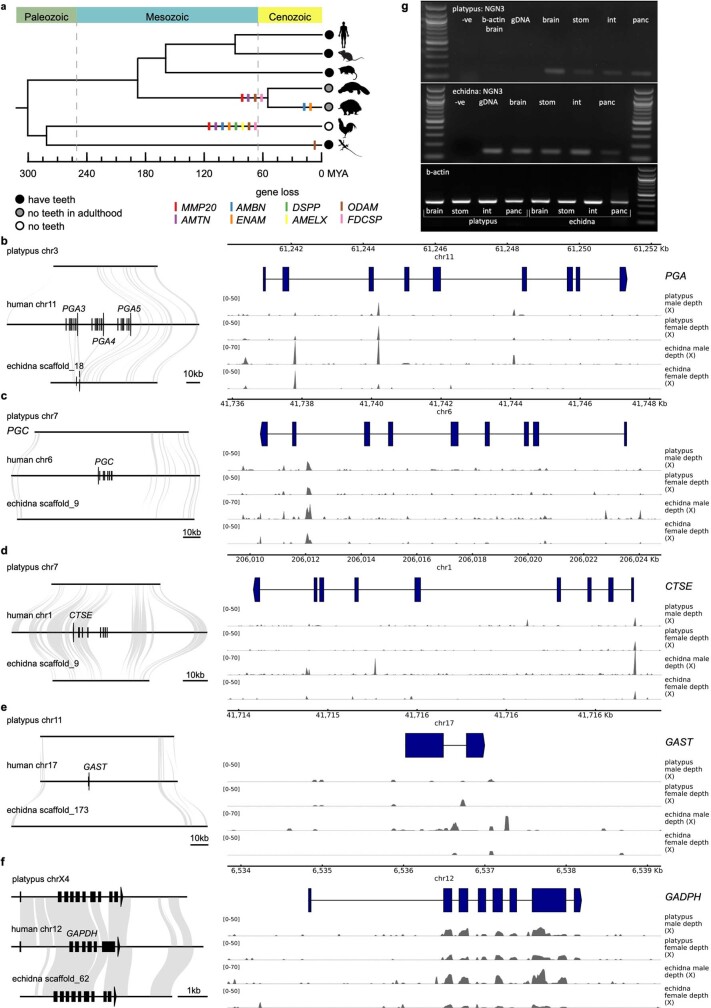
Loss of dietary-related genes in monotremes. **a**, Tooth-related gene loss in representative mammals and reptiles. **b**–**f**, Potential loss of digestion-related genes in both monotremes shown by whole-genome alignment and read mapping. In each panel there are three lines in the synteny plot, representing the orthologous region of the genes in platypus, human and echidna from top to bottom, respectively. Grey links indicate human–platypus and human–echidna LASTZ alignments. Each rectangle or triangle represents an exon. Fragmented genes are marked by dashed lines. Illumina reads of platypus and echidna are aligned to the platypus or human genome (Ensembl release 87) and the flanking region of each gene is visualized by pyGenomicTrack. *GAPDH* region is also plotted as a control. **g**, RT–PCR expression analysis shows expression of *NGN3* in brain, stomach, intestine and pancreas of both platypus and echidna. These results are similar to other mammals. This, together with sequencing results, shows that *NGN3* in monotremes is present and is likely to be functioning normally. NGN3, NGN3 primers; b-actin, β-actin primers; -ve, negative control, no template; gDNA, genomic DNA template; brain, brain cDNA template; stom, stomach cDNA template; int, intestine cDNA template; panc, pancreas cDNA template. Lanes 1 (top), 1, 8 (middle) and 9 (bottom) are a 100-bp DNA ladder: 1,517, 1,200, 1,000, 900, 800, 700, 600, 500/517, 400, 300, 200 and 100 bp. Expected sizes of PCR products for *NGN3* in platypus is 157 bp and for echidna 145 bp, and the PCR product for the β-actin genomic region is 597 bp and cDNA is 348 bp. Silhouettes of human and opossum are from https://www.flaticon.com/. The silhouette of the platypus is created by S. Werning and is reproduced under a Creative Commons Attribution 3.0 Unported licence (http://creativecommons.org/licenses/by/3.0/).

Platypuses consume aquatic invertebrates whereas echidnas feed predominantly on social insects. Although the recent ancestor of monotremes had adult teeth, both extant monotremes lack teeth^23^. Of eight genes involved in tooth development^24^, four genes were lost in both monotreme genomes, suggesting that the loss occurred in their recent common ancestors (Extended Data Fig. 8a and Supplementary Table 42), consistent with other toothless or enamel-less eutherians^25^. Echidnas (but not platypuses) further lost two enamel genes. Analysis of genes involved in stomach function revealed that the considerable loss of digestive genes (reported in platypus^26^) is shared with echidna and probably occurred in the monotreme ancestor, although *NGN3*—which is essential for stomach and pancreas development—has been maintained in both species (Extended Data Fig. 8b–g and Supplementary Table 43).

**Fig. 4:**
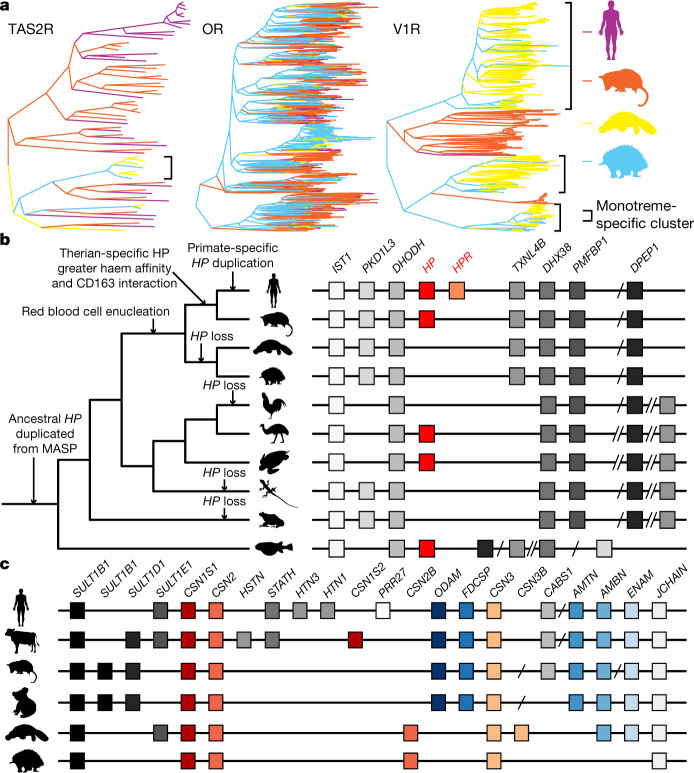
Genomic features related to biological characteristics of the monotremes. **a**, Differences in numbers of TAS2R, OR and V1R genes between platypus and echidna. **b**, Phylogeny and synteny of the *HP* gene. Regions are not drawn to scale. **c**, Synteny conservation of the region surrounding caseins (CSN genes) and the ancestral teeth genes (*ODAM*, *FDCSP*, *AMTN*, *AMBN* and *ENAM*). Silhouettes of the human, opossum, koala and frog are from https://www.flaticon.com/. Silhouettes of the platypus and Tasmanian devil are created by S. Werning and the emu silhouette is created by D. Naish (vectorized by T. M. Keesey); all three silhouettes are reproduced under the Creative Commons Attribution 3.0 Unported licence (http://creativecommons.org/licenses/by/3.0/).

**Extended Data Fig. 9:**
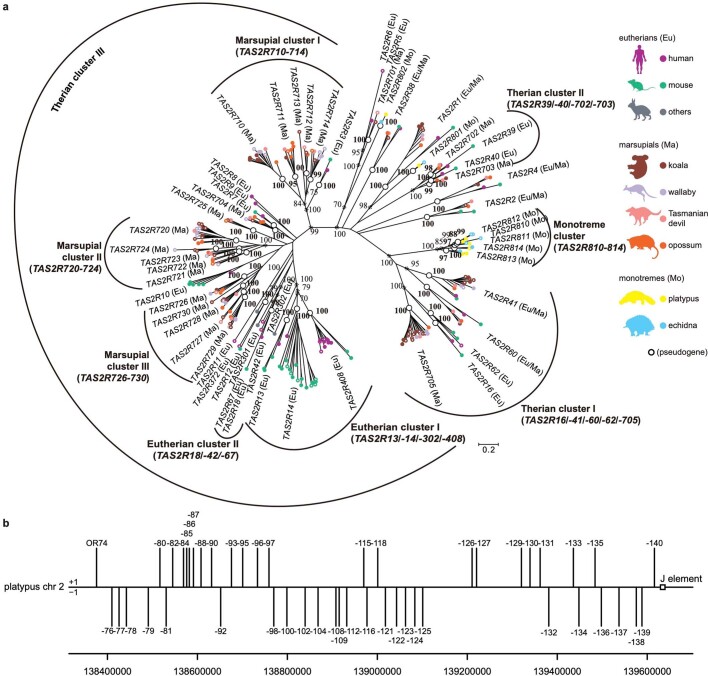
Taste-receptor evolution and olfactory-receptor organization in monotremes. **a**, Maximum-likelihood mammalian-wide gene tree of the bitter taste receptors (TAS2R genes). There are 28 eutherian (Eu), 27 marsupial (Ma) and 7 monotreme-specific (Mo) orthologous gene groups (supported by ≥95% bootstrap values), where the nodes of orthologous gene group clades are indicated by white open circles. Bootstrap values of ≥70% in the nodes connecting orthologous gene group clades are indicated by asterisks. There are 3 therian (I, II and III), 2 eutherian (I and II), 3 marsupial (I, II and III) and one monotreme-specific clusters in which massive expansion events occurred in the common ancestor of each taxon after the split from its previous ancestors. **b**, Genomic organization of the intact class I olfactory receptor (OR) cluster spanning over 1.2 Mb on platypus chromosome 2 (138,375,798–139,616,970 bp). The vertical lines indicate the 48 intact class I OR genes. The white open box indicates the J element, a presumable *cis*-regulatory element (enhancer) for the mammalian class I OR cluster (chromosome 2: 139,639,465–139,639,907 bp). Silhouettes of human, opossum and koala are from https://www.flaticon.com/. Silhouettes of the platypus and Tasmanian devil are created by S. Werning and are reproduced under a Creative Commons Attribution 3.0 Unported licence (http://creativecommons.org/licenses/by/3.0/).

Chemosensory systems mediate animal behaviour that is essential for survival and reproduction through the direct interaction with environmental chemical cues^27^. For example, eutherian mammals have more than 25 copies of bitter taste receptor genes (*TAS2R* genes)^27,28^, whereas this gene family is considerably smaller in monotremes (Extended Data Fig. 9a) with only 7 in platypus (Supplementary Tables 44, 45). The number is reduced to three in echidna (Fig. 4a and Supplementary Results). This reduction is also observed in pangolins, which suggests convergent evolution that results from the insectivore diet of both echidnas and pangolins^29^.

The nasal cavity of the platypus is closed off during diving and the size of the main olfactory bulb of the platypus is much smaller than that of the echidna^1^. Consistent with this, the number of olfactory receptors (OR genes) in platypus (299) is much smaller than in echidna (693) (Fig. 4a and Supplementary Table 46). The difference in the large olfactory bulb and OR repertoire in echidna may contribute to the ability to search for odours of underground prey, whereas the platypus relies on electroreception to detect prey in the water. However, the size of the accessory olfactory bulb is larger in the platypus than in the echidna^1^. The accessory olfactory bulb receives projections from the vomeronasal organ, and there is a marked expansion of the number of vomeronasal type-1 receptors (V1R genes) in the platypus (262) compared with the echidna (28) (Fig. 4a and Supplementary Table 47). Vomeronasal receptors probably have important roles in courtship, parental care, induction of lactation and milk ejection in monotremes^23^. Therefore, the diversification of the olfactory bulb and accessory olfactory bulb systems in monotremes provide an interesting example of the eco-evolutionary trade-off. V1R amplification has been associated with the size of the vomeronasal organ and nocturnal activity^30^. This is also consistent with the fact that the platypus closes its eyes when diving and therefore relies entirely on other senses underwater and in the burrow.

## Haemoglobin degradation in monotremes

The semi-aquatic lifestyle of the platypus is supported by particularly high haemoglobin levels and large numbers of small red blood cells^31^. The haemoglobin–haem detoxification system in mammals provides efficient clearance to minimize oxidative damage^32^ in which haptoglobin is the haemoglobin chaperone^32^ and free haem is bound by haemopexin and alpha-1 microglobulin^33^.

**Extended Data Fig. 10:**
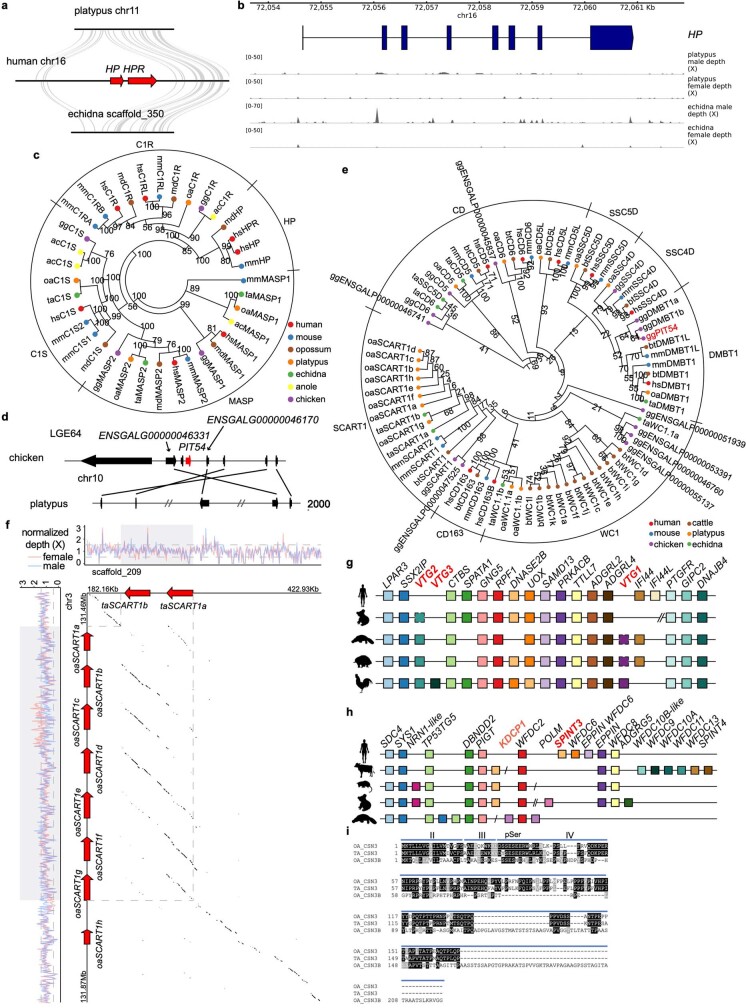
Genomic features related to haemoglobin clearance and reproduction in monotremes. **a**, **b**, Confirmation of *HP* absence in monotremes by whole-genome alignment (**a**) and read mapping (**b**). Grey links indicate human–platypus and human–echidna LASTZ alignments. Illumina reads of platypus and echidna are aligned to the human genome (Ensembl release 87) and coding regions of *HP* are visualized by pyGenomicTrack. Limited coverage is found at the exons of *HP*, suggesting the absence of *HP* in monotremes. **c**, Phylogenetic tree of HP and related proteases across different species using the maximum-likelihood method. Node IDs are in format of ‘species geneID’. Branch length is not shown here. **d**, Gene synteny plot of the *PIT54* region between chicken and platypus. Echidna is not shown in the figure as the flanking orthologues of *PIT54* are on different scaffolds, preventing us from determining the presence of the gene by synteny. **e**, Phylogenetic tree of members of the group B scavenger receptor cysteine-rich family across different species using the neighbour-joining method. Gene IDs are formatted as ‘species geneID’. Branch length is not shown here. **f**, Confirmation of *SCART1* number difference by dot plot and mapping depth of *SCART1* orthologous regions between platypus and echidna. The region of the *SCART1* cluster in platypus is plotted along the *x* axis while the sequence of echidna is plotted along the *y* axis. Lines in dot plot are visualized according to LASTZ alignment between the two species. Normalized male and female read depths along each sequence is calculated in 500-bp windows, and plotted along each sequence. Normalized depth of both sexes, especially those in the shading region, is centred at 1 along both species, confirming the *SCART1* number difference between the two species is true and is not due to assembly issues. **g**, Synteny conservation of vitellogenin genes. Synteny conservation of the region surrounding the vitellogenin (*VTG*) genes *VTG1*, *VTG2* and *VTG3*. Pseudogenes are marked by a dashed outline. Monotremes have pseudogene *VTG1*, functional *VTG2* and no *VTG3*; and there is a pseudogene *VTG2* in koala. Syntenic maps are shown for human (*Homo sapiens*), koala (*Phascolarctos cinereus*), chicken (*Gallus gallus*), platypus (*O. anatinus*) and echidna (*T. aculeatus*). Koala scaffold 1, NW_018343984.1; koala scaffold 2, NW_018344134.1. Gene distances are not to scale. **h**, Synteny conservation of regions containing *SPINT3*. Synteny conservation of the region surrounding serine peptidase inhibitor, Kunitz-type, 3 (*SPINT3*). No copy of *SPINT3* is detected in platypus but many of the other flanking genes in the region are conserved. Other members with a WFDC domain are detected including two Kunitz-domain members that did not align to any known gene (labelled *KDCP1*). Syntenic maps are reported for human (*H. sapiens*), cow (*B. taurus*), grey short-tailed opossum (*Monodelphis domestica*), koala (*P. cinereus*) and platypus (*O. anatinus*). Koala scaffold 1, NW_018343967.1; koala scaffold 2, NW_018344098.1. Gene distances are not to scale. **i**, Casein 3 (CSN3) protein sequence alignment in monotremes. All three CSN3 proteins identified in the monotremes have the classic five-exon structure of CSN3 with the untranslated exons I and IV (not shown), the signal peptide in exon II, a small exon III coding for 11 residues, a pSER cluster (S**) at the 5′ end of exon IV and a relatively large P/Q-rich exon IV. OA, *O. anatinus* (platypus); TA, *T. aculeatus* (short-beaked echidna). Silhouettes of human, opossum and koala are from https://www.flaticon.com/. The silhouette of the platypus is created by S. Werning and is reproduced under a Creative Commons Attribution 3.0 Unported licence (http://creativecommons.org/licenses/by/3.0/).

Both the haemopexin and alpha-1 microglobulin genes are found in the monotreme genomes, whereas the haptoglobin gene is absent (Fig. 4b, Extended Data Fig. 10a, b and Supplementary Table 48), which suggests that monotremes evolved a haemoglobin clearance system that is different from that of other mammals. Haptoglobin evolved in the common ancestor of vertebrates from an immune gene of the MASP family^33^ but has neofunctionalized in mammals to bind to haemoglobin with a higher affinity and to bind to the CD163A receptor, which is also absent in monotremes, for clearance in macrophages^34^. The absence of the haptoglobin gene and *CD163A* in monotremes suggests that the neofunctionalization of haptoglobin happened after the divergence of monotremes from therians, not before it as previously thought^34^, and long after the evolution of enucleated red blood cells in the common ancestor of mammals^35^. Several nonmammalian vertebrates have lost haptoglobin, including chicken^34^ (Fig. 4b), in which an alternative, secreted CD163 family member, *PIT54*, is the haemoglobin-binding chaperone^33^. Phylogenetic analysis shows that monotremes lack genes that cluster with haptoglobin in the MASP family or a *PIT54* orthologue (Extended Data Fig. 10c–e and Supplementary Table 50). We confirmed the expansion of the CD163 family in platypus^2^ (ten members) and found five in echidna, compared with two and three in humans and mice, respectively (Extended Data Fig. 10e, f). As mammalian CD163A can bind to haemoglobin in the absence of haptoglobin^36^ and one CD163 family member has become the haemoglobin chaperone in chicken, the CD163 family protein(s) may have evolved this role in monotremes.

## Transition from oviparity to viviparity

Monotremes provide the key to understanding how viviparity evolved in mammals. They are not as dependent on egg proteins as egg-laying avian and reptilian species owing to their nutrient acquisition from uterine secretions^23,37^, and the subsequent reliance of the young on lactation. Whereas reptiles have three functional copies of the major egg protein vitellogenin (*VTG*)^38^, in monotremes we found only one functional copy (*VTG2*) (Extended Data Fig. 10g and Supplementary Table 52) and a partial sequence for *VTG1*.

Similar to marsupials, monotremes have an extended lactation period and the composition of the milk changes dynamically as the development progresses to match the changing needs of the young^37^. SPINT3, a major milk-specific protein that is present in early lactation of therians with a probable role in the protection of immunoincompetent young in marsupials^39^, is absent in monotremes. Syntenic analysis confirmed that this region is conserved in platypus but contains two copies of a new protein that contains a Kunitz domain (Extended Data Fig. 10h and Supplementary Table 53). The Kunitz family is a rapidly evolving family, and one of the new members could have a immunoprotective function similar to *SPINT3* in monotremes.

The monotreme genomes contain most of the milk genes that have been identified in therian mammals^38,40^. Most mammals have three casein genes^41^, which encode the most abundant milk proteins secreted throughout lactation (Fig. 4c). In addition to these genes, monotremes have extra caseins that are not found in therian mammals, with unknown functions, an extra copy of *CSN2* (*CSN2B*) (previously reported^40^) and *CSN3* (*CSN3B*) in platypus (described here), which has the classic structure of *CSN3*^42^ (Extended Data Fig. 10i and Supplementary Table 54).

All caseins are members of the secretory calcium-binding phosphoprotein (SCPP) gene family and are thought to have evolved from other SCPP genes, namely the teeth-related gene *ODAM* through its derivatives *FDCSP* and *SCPPPQ1*^42^. As reported above (see ‘Eco-evolutionary adaptation of diet’), extant monotremes appear to have lost both *ODAM* and *FDCSP*. Syntenic analysis showed that the additional monotreme casein genes (*CSN2B* and *CSN3B*) are found in the same therian chromosomal region as *ODAM* and *FDCSP* and within the casein locus (Fig. 4c), providing further evidence that caseins evolved from odontogenic genes.

## Summary

Complete and accurate reference genomes and annotations are critical for evolutionary and functional analyses. It remains a challenge to produce a highly accurate chromosome-level assembly, particularly for differentiated sex chromosomes. We have produced a high-quality platypus genome using a combination of single-molecule sequencing technology and multiple sources of physical mapping methods to assign most of the sequences to a chromosome-scale assembly. This permits better-resolved analyses of the origin and diversification of the complex sex chromosome system that evolved specifically in monotremes. We delineate ancient and lineage-specific changes in the sensory system, haemoglobin degradation and reproduction that represent some of the most fascinating biology of platypus and echidna. The new genomes of both species will enable further insights into therian innovations and the biology and evolution of these extraordinary egg-laying mammals.

## Methods

### Data reporting

No statistical methods were used to predetermine sample size. The experiments were not randomized and the investigators were not blinded to allocation during experiments and outcome assessment.

### Ethics and sample collection

Pmale08, Pmale09 and Emale12 were collected under AEC permits S-49-2006, S-032-2008 and S-2011-146 at Upper Barnard River (New South Wales, Australia) during the breeding season. Emale01 was collected under San Diego Zoo Global IACUC approval 18-024 and vouched at San Diego Natural History Museum.

### Sequencing and assembling

Skeletal muscle of Pmale09 was used for PacBio, 10X and BioNano genome sequencing and the liver of Pmale09 was used for Hi-C (Phase Genomics); the liver of Pmale08 was used for Chicago Hi-C (Dovetail Genomics). Heart muscle of Emale01 was used for a variety of library construction and Illumina sequencing analyses. Muscle of Emale12 was used for 10X and BioNano genome sequencing and liver of Emale12 was used for Hi-C (Phase Genomics). Echidna RNA was extracted from brain, cerebellum, kidney, liver, testis and ovary and sequenced using a previously published procedure^43^. Platypus Y chromosome BAC isolation via hybridization was performed using a previous published procedure^4^ and sequenced with PacBio. The platypus genome was assembled following VGP assembly pipeline v.1.0. The echidna genome was assembled using Platanus^44^ (v.1.2.1) and followed by three steps of scaffolding in the order of 10X, BioNano and Hi-C. Manual curation was performed for both assemblies. Details are available in the Supplementary Methods.

### Sex-borne sequence identification

Female and male reads were mapped to the genome using BWA ALN^45^ (v.0.7.12). The read depth of each sex was calculated in 5-kb non-overlapping windows to identify X-borne sequences and 2-kb non-overlapping windows to identify Y-borne sequences, normalized against the median depth. To identify X-borne sequences, we calculated the female-to-male (F/M) depth ratio of regions that were covered by both sexes in each scaffold, requiring a minimum coverage of 80%, and assigned sequences to X-borne if the depth ratio ranged between 1.5 and 2.5. To identify Y-borne sequences, we calculated the F/M depth ratio as well as the F/M coverage ratio and assigned scaffolds to Y-borne if either ratio was within the range of 0.0–0.3. Parameter evaluation details are available in the Supplementary Methods.

### Chromosome anchoring

We collected 75 BAC and 179 marker genes (Supplementary Table 3) and ordered them according to their relative order from those papers. Protein sequences of the gene markers were compared to mOrnAna1 using TBLASTN and the best hit was kept, after which the markers were analysed using GeneWise^46^ (v.2.4.1) to obtain the location within a scaffold. BAC-end reads were mapped to the assembly using BWA MEM^47^ (v.0.7.12) and the best hits were kept. We also used the anchored sequences of OANA5 except for the sequence of chromosome 14 to anchor the scaffolds into chromosomes. Scaffolds were orientated and ordered first based on the order of FISH or gene markers then on the order in OANA5. All identified PARs were included in chromosome X. We collected assembled Y contigs from a previously published study^4^ and generated some Y-BAC PacBio sequencing data. Assembled Y contigs were mapped to the platypus assembly using BWA MEM and Y-BAC PacBio reads were mapped using minimap2 (v.2.13)^48^. As evidence of both Y2 and Y3 were found on scaffold_229_arrow_ctg1 and scaffold_269_arrow_ctg1 and the covered regions overlapped, these two scaffolds were excluded from the chromosome Y classification. Classified Y-borne scaffolds failed to anchor and orient due to the lack of information. We also curated and anchored some echidna X-borne scaffolds to chromosome X based on Mashmap^49^ (v.2.0) one-to-one results with platypus^50^.

### Annotation

We identified repetitive elements in both assemblies using the same pipeline, which included homologue-based and de novo prediction. For the homology-based method, we used default repeat library from Repbase (v.21.11)^51^ for RepeatMasker (v.4.0.6)^52^, trf (v.4.07)^53^ and Proteinmasker (v.4.0.6)^52^ to annotate. For the de novo method, we first ran RepeatModeler (v.1.0.8) to construct the consensus sequence library for each monotreme using their genome as input, then aligned the genome against each consensus library to identify repeats using RepeatMasker. Gene annotation was performed by merging the homology, de novo prediction and transcriptome analyses to build a consensus gene set of each species. Protein sequences from human, mouse, opossum, platypus, chicken and green lizard (*Anolis carolinensis*) from Ensembl^54^ (release 87) were aligned to the genome using TBLASTN^55^ (v.2.2.26) (*e* < 1 × 10^−5^). Candidate gene regions were refined using GeneWise for more accurate gene models. We randomly selected 1,000 high score homology-based genes to train Augustus^56^ (v.3.0.3) for de novo prediction on a repeat N-masked genome. We also mapped RNA-sequencing reads of the platypus from a previously published study^57^ and echidna to their respective assemblies using HISAT2^58^ (v.2.0.4), and constructed transcripts using stringTie^59^ (v.1.2.3). Results from these three methods were merged into a nonredundant gene set. Possible retrogenes were filtered according to their hit to SwissProt database^60^ (release 2015_12) or Iprscan^61^ (v.5.16-55.0). We used the SwissProt database (*e* < 1 × 10^−5^) to annotate the function of the genes. Iprscan was used to annotate the GO of genes. Detailed descriptions of the manual annotation, curation and phylogenetic analysis of genes related to imprinting, immune system, reproduction and haemoglobin degradation can be found in the Supplementary Methods.

### Gap analysis

We identified gap-filling regions using an alignment-based strategy similar to a previously published study^62^. We considered gaps for which both flanking regions mapped to mOrnAna1 as closed gaps. Only properly closed gaps defined by (1) both flanking regions were aligned but did not overlap and (2) closed gap size were within 100 times the estimated gap size in OANA5 were considered for repeat and gene improvement analysis.

### Redundant sequences analysis

We performed two rounds of Mashmap with parameters ‘-f one-to-one -s 2000’ using mOrnAna1 as reference and OANA5 as query. A one-to-one relationship was obtained in the first round of Mashmap. In the second round of Mashmap, those OANA5 sequences that were unmapped in the first round of mapping were used as query. Candidate redundant sequences were obtained from the second round Mashmap result, but excluded regions that were gaps in OANA5. Female and male reads were then mapped to OANA5 and mOrnAna1 using BWA ALN and normalized by the mode depth.

### Gene set comparison

We performed LASTZ^63^ (v.1.04.00) alignment using OANA5 as reference with parameter set ‘--hspthresh=4500 --gap=600,150 --ydrop=15000 --notransition --format=axt’ and a score matrix for the comparison of closely related species to generate a chain file for gene location liftover from OANA5 to mOrnAna1. Gene coordinates in OANA5 were first converted to mOrnAna1 using in-house-generated scripts with the chain file. We searched for overlap between the converted OANA5 gene set and mOrnAna1 gene set. Fragmented genes were defined as multiple converted OANA5 genes that overlapped with a single mOrnAna1 gene. A one-to-one gene pair between the two gene sets was defined as the liftover of the OANA5 gene when it overlapped with only one mOrnAna1 gene. Only one-to-one pairs were used for the comparison of open-reading frame completeness. We defined a gene as having a complete open-reading frame if its first codon is a start codon and the last codon is a stop codon.

### Identification of one-to-one orthologues and synteny blocks between the human sequence and sequences of other species

We defined one-to-one orthologues between the human sequence and the sequences of other species by considering both reciprocal best BLASTP hits (RBH) and synteny, taking the human sequence as reference, as previously described^64^. First, we conducted BLASTP for all protein sequences from human and other species including mouse, opossum, platypus, echidna, chicken and green lizard with an *e*-value cut-off of 1 × 10^−7^, and combined local alignments with the SOLAR (http://treesoft.svn.sourceforge.net/viewrc/treesoft/). Next, we identified RBH orthologues between human and every other species on the basis of the following parameters: alignment score, alignment rate and identity. From these RBH orthologues, we retained those pairs with conserved synteny across species. Synteny was determined based on their flanking genes. If RBH orthologous gene pairs shared the same flanking genes, we retained the genes for downstream analyses. Finally, we merged pairwise orthologue lists according to the human coordinates. In this way, we produced the final one-to-one orthologue set across species.

We used the human genome as the reference and aligned it with other species using LASTZ with parameter set ‘--hspthresh=4500 --gap=600,150 --ydrop=15000 –notransition --format=axt’ and a score matrix for the comparison of closely related species. Alignments were converted into ‘chain’ and ‘net’ results with different levels of alignment scores using utilities of the UCSC Genome Browser (http://genomewiki.ucsc.edu/index.php/), and the pairwise synteny blocks between genomes of each species and the human genome were extracted according to the net result. Only alignments larger than 10 kb were kept. The synteny blocks were further cleaned of overlapping genes. N50 and the total length of the synteny block inferred from each human–species pair were calculated based on the human coordinates.

### Phylogenetic analysis

The phylogenetic tree was constructed using concatenated four-degenerated sites from the 7,946 one-to-one orthologues using RaxML^65^ (v.8.2.4) with parameter set ‘-m GTRCAT -# 100 -p 12345 -x 12345 -f a’ and chicken and green lizard were specified as the outgroup. MCMCtree in PAML^66^ (v.4.7) was used to estimate divergence time of each species with calibration points obtained from a previously published study^67^ using the same data. Points and time range included the most recent common ancestor of human–mouse, 85–94 million years ago; human–opossum 150–167 million years ago; human–platypus, 163–191 million years ago, human–chicken, 297–326 million years ago, anole–chicken, 276–286 million years ago. The seed used for MCMC was 1192664277.

### Substitution rate analysis

We first performed pairwise whole-genome LASTZ alignment using 12 mammals (*Macaca mulatta*, *Tupaia belangeri*, *Mus musculus*, *Canis lupus familiaris*, *Myotis lucifugus*, *Bos taurus*, *Sorex araneus*, *Loxodonta africana*, *Dasypus novemcinctus*, *Monodelphis domestica*, *O. anatinus* and *T. aculeatus*) with the human genome as the reference genome, with the parameter set ‘--step=19 --hspthresh=2200 -inner=2000 --ydrop=3400 --gappedthresh=10000 --format=axt’ and a score matrix for the comparison of distantly related species. Pairwise alignments were merged using MULTIZ^68^ (v.11.2). The four-degenerated site alignment was extracted based on the human gene set (Ensembl release 87), concatenated and fed to phyloFit in the PHAST package^69^ (v.1.5) for the calculation of branch lengths (substitution per site). The substitution rate was calculated by dividing the branch length to the mammalian common ancestor to the mammal–reptile divergence time.

### Gene family analysis

Gene families across the seven species were generated using orthoMCL^70^ (v.2.0.9) with BLASTP results (*e* < 1 × 10^−7^) and was fed to CAFÉ^71^ (v.4.2) along with the phylogenetic tree. We first estimated the assembly error by excluding families with more than 100 members. Then the estimated rate was used to infer the family size at every node for each family. The ancestral node gene number of families with more than 100 members among extant species were inferred separately. We extracted genes based on the human gene set for GO enrichment (*χ*^2^ test) of the significantly expanded family (Viterbi *P* < 0.05) for the mammalian ancestor. A false-discovery rate (FDR) adjustment was used for multiple-test corrections in GO enrichment analyses.

### Mammalian-specific highly conserved element analysis

We used the same MULTIZ alignment of the substitution rate analysis and identified mammalian-specific highly conserved elements (MSHCEs) using a similar strategy as has previously been described^72^. At least 80% of species and at least one species in eutherians, marsupials and monotremes were required to be present in alignments. Type-I MSHCEs were defined as HCEs to which no outgroup could be aligned; type-II MSHCEs were HCEs that were significantly conversed (*P* < 0.01) in mammals compared to mammals + outgroup calculated using phyloP (Benjamini–Hochberg adjusted). We considered four sets of outgroup combinations: (1) green lizard only; (2) chicken only; (3) two reptiles and one frog; and (4) two reptiles, one frog and one fish, and only kept those that were significantly conserved in all four sets of statistical tests (Benjamini–Hochberg adjusted *P* < 0.01). Only elements ≥20 bp were kept for further analysis.

To annotate MSHCEs to possible functional elements, we used the human annotation (Ensembl release 87) as a reference and classified the elements into the coding sequence, 5′ and 3′ untranslated regions, non-coding RNA, pseudogene, intron, upstream 10-kb region (from start codon), downstream 10-kb region (from the stop codon) and intergenic regions, with the same hierarchical order if the regions overlapped. Genes located within the upstream or downstream 10-kb range of MSHCEs were considered to be MSHCE-associated genes, and ordered by the length of the element. The top-300 MSHCE-associated genes were used in the GO enrichment analysis (*χ*^2^ test, FDR-adjusted) and visualized using REVIGO^73^.

### Mammalian karyotype reconstruction

We used pairwise LASTZ alignments of the opossum, Tasmanian devil, platypus, chicken and common wall lizard (*Podarcis muralis*) genomes to the human genome as input. Echidna was not used here as most of the sequences were not anchored to chromosomes, which would lead to a more fragmented reconstruction. With the net and chain results, conserved segments that were uniquely and universally presented in all six species were obtained using inferCARs^74^ (release 2006-Jun-16). Marsupial and therian ancestral karyotypes were inferred using ANGES^75^ (v.1.01) using the branch-and-bound algorithm, and the resulting continuous ancestral regions (CARs) were further reorganized based on the previously predicted configuration^76^ (Supplementary Tables 22, 23). We replaced the conserved segments of the human, opossum and Tasmanian devil genomes with those of the reconstructed therian ancestral karyotype and reconstructed marsupial ancestral karyotype using ANGES with the same parameters except setting the target reconstruction node to mammalian ancestor. We reorganized CARs on the basis of gene synteny among ingroups and outgroups inferred using MCScanX^77^ (release 08-05-2012), requiring that there is synteny across CARs in at least one ingroup–outgroup pair (Supplementary Tables 22, 23). Pairwise MCScanX was run among the six species with BLASTP (*e* < 1 × 10^−7^).

Rearrangement events in each lineage were inferred using GRIMM^78^ (v.2.1) by taking the karyotypes of the most recent ancestor and the child as input. The breakpoint number in each lineage was calculated on the basis of the output of GRIMM using an in-house-generated script, in which one breakpoint was counted in fission, two breakpoints were counted in translocation, and one or two breakpoints were counted in inversion, depending on whether the inversion happened at the end of the chromosome. Calculations were done using resolutions of 500 kb and 300 kb, and using the raw ANGES output and reorganized output, respectively (Supplementary Table 28). Differences in breakpoint rates compared to the average of all branches were tested as previously described^79^.

### Gametologue identification

We used BLASTP to compare all Y-borne genes to all X-borne genes (*e* < 1 × 10^−5^) and kept the best hit for each Y-borne gene. Candidate gametologue pairs were further confirmed if both of the genes were mapped to the same gene in NCBI or the SwissProt database. Four gametologues (platypus *AMHX* and *FEM1CX* from OANA5, and *SDHAY* and *HNRNPKY* from ref. ^14^) were added as they were missing in mOrnAna1. Translated genes were aligned using PRANK^80^, filtered using Gblock^81^, and converted back into the alignment of the coding sequence. d*S* was calculated using codeml in PAML with ‘runmode=-2’.

### Demarcate evolutionary strata

We aligned all platypus Y-borne scaffolds (N-masked) to all platypus X-borne sequences (N-masked), and aligned all echidna Y-borne scaffolds (N-masked) to all echidna X-borne sequences (N-masked), using LASTZ with the parameter set ‘--step=19 --hspthresh=2200 --inner=2000 --ydrop=3400 --gappedthresh=10000 --format=axt’ and a score matrix set for the comparison of distantly related species. On the basis of the net and ‘maf’ results, the identity of each alignment block was calculated in a 2-kb non-overlapped window and the aligned Y-borne sequences were oriented along the X chromosomes. Identity along X chromosomes was colour-coded for visualization.

### Expression calculation

RNA-sequencing reads of platypus (SRP102989) and echidna were mapped to the genome using HISTA2. Uniquely mapped reads were used in the calculation and normalization of the reads per kilobase per million reads (RPKM) using DESeq^82^ (v.1.28.0) to generate an expression matrix for each species. For tissues that were available in both sexes, we computed the median RPKM of each X-borne gene, and computed its F/M RPKM ratio (requiring RPKM in both sexes to be ≥1) to determine dosage-compensation status. We used the median expression value in each tissue to calculate the tissue specificity index TAU^83^ for each gene. We defined tissue-specific expression as a gene that shows at least twofold higher expression in tissue with the highest expression than in any other tissue, the highest RPKM > 1 and TAU > 0.8.

### Building genome-wide Hi-C interaction maps

Genome-wide interaction maps at a 100-kb resolution were generated for platypus, echidna and human (SRX641267) with HiC-Pro^84^ (v.2.10.0). For echidna, we only retained scaffolds >10 kb as the large number of short scaffolds would cause ICE normalization failure. The normalized sex chromosomes submatrix was extracted for quantification and plotting with ggplot2 (v.3.2.1). For human, we used the scaled homologous sequences of platypus for quantification and plotting.

### Identification of TADs and CTCF-binding sites

HiC-Pro interaction maps were transformed to h5 format using hicConvertFormat and fed to hicFindTADs with the parameters ‘--outPrefix TAD --numberOfProcessors 32 --correctForMultipleTesting fdr’ to identify TADs with HiCExplorer^85^ (v.3.0). The human CTCF motif^86^ was used as a bait by fimo in MEME^87^ (v.4.12.0) to identify putative CTCF-binding sites. CTCF densities in every 100 kb non-overlapping sliding window along the platypus sex chromosomes or scaled homologous sequences of echidna, human and chicken were compared.

### FISH

BACs were obtained from the Children’s Hospital Oakland Research Institute from the platypus BAC library CH236: CH236-775N6 13q2; CH236-97I3 15p1 and CUGI BAC/EST resource centre from the platypus BAC library Oa_Ab: Oa_Bb-155A12 autosomal (*WSB1*); Oa_Bb-145P09 Y2; Oa_Bb-397I21 Y3. The Super_Scaffold_40-specific probe was amplified from platypus genomic DNA. Gene ENSOANT00000009075.3 was amplified using primers GTCTAAAGACAAGTGTACATCTGTGAC and GTGACTTCTCTTGCGAACACAC. The 3.9-kb product was cloned into pGEM-T Easy (Promega). BAC probes were directly labelled with dUTP Alexa Fluor 594-dUTP, aminoallyl-dUTP-XX-ATTO-488 (Jena Bioscience) using the Nick Translation Kit (Roche Diagnostics) and the Super_Scaffold_40-specific probe labelled with biotin using the Biotin-Nick Translation Mix (1175824919, Roche Diagnostics). The FISH protocol was carried out on cultured fibroblasts from platypus (authenticated by karyotype, not mycoplasma tested) obtained from animals captured at the Upper Barnard River (New South Wales, Australia) during the breeding season (AEC permits S-49-2006, S-032-2008 and S-2011-146) as previously described^88^ with the following exceptions. Slides were denatured at 70 °C for 3 min in 70% formamide in 2× SSC, 1 mg DNA probe was used per slide, pre-annealing of repetitive DNA sequences was done at 37 °C for 30–60 min. Detection of biotin-labelled probes was done using Rhodamine Avidin D (Vector Laboratories, A-2002), goat Biotinylated anti-avidin D (Vector Laboratories, BA-0300) and Rhodamine Avidin D. Slides were blocked in 4 × SSC, 1% BSA fraction V, for 30 min at 37 °C. Rhodamine Avidin D and Biotinylated anti-avidin D and the second Rhodamine Avidin D were diluted in 4 × SSC, 1% BSA fraction V and were incubated on slides for 45 min at 37 °C, after each step washes were done in 4 × SSC, 4 × SSC, 0.1% triton, 4 × SSC at room temperature for 10 min each. Slides were mounted in VECTASHIELD with DAPI (Vector Laboratories, H-1200). Sample size was determined according to ref. ^89^, but was limited by material availability. Images were captured on a Nikon Ti Microscope using NIS-Elements AR 4.20.00 software and processed with ImageJ (v.2.0.0). Fisher’s exact test was performed with matrix containing mean of associated and non-associated cells from the three replicates. No blinding nor randomization was performed.

### Reporting summary

Further information on research design is available in the Nature Research Reporting Summary linked to this paper.

## Online content

Any methods, additional references, Nature Research reporting summaries, source data, extended data, supplementary information, acknowledgements, peer review information; details of author contributions and competing interests; and statements of data and code availability are available at 10.1038/s41586-020-03039-0.

## Supplementary information

Supplementary Information Details on the sample collection and analytical methods used in this study. Also includes Supplementary Results with the detailed analyses results, as well as Supplementary Tables 1, 2, 6, 7, 9, 13-16, 18, 21, 24, 25, 34, 38, 41-45 and 50.

Reporting Summary

Supplementary Tables This file includes Supplementary Tables 3-5, 8, 10-12, 17, 19, 20, 22, 23, 26-33, 35-37, 39, 40, 46-49 and 51-54.

## Data Availability

The platypus whole-genome shotgun project has been deposited at GenBank (project accessions PRJNA489114 and PRJNA489115), CNSA (https://db.cngb.org/cnsa/) of CNGBdb (accession CNP0000130) and GenomeArk (https://vgp.github.io/genomeark-curated-assembly/Ornithorhynchus_anatinus/). The echidna whole-genome shotgun project has been deposited at GenBank (project accession PRJNA576333), CNSA of CNGBdb (accession CNP0000697) and GenomeArk at (https://vgp.github.io/genomeark/Tachyglossus_aculeatus/). Echidna RNA-sequencing data have been deposited at GenBank (project accession PRJNA591380) and CNSA of CNGBdb (accession CNP0000779). Public database used in this study include: NCBI (https://www.ncbi.nlm.nih.gov/), Ensembl (release 87) (http://dec2016.archive.ensembl.org/index.html), Uniprot (https://www.uniprot.org/) and Repbase (https://www.girinst.org/repbase/). Accession codes of genes are available in Supplementary Tables 31, 33, 37, 49, 51.

## Code availability

In-house-generated scripts used in this study are shared on GitHub (https://github.com/ZhangLabSZ/MonotremeGenome).
