# Supplementary material for: Platypus and echidna genomes reveal mammalian biology and evolution
Source: Nature. 2021 Jan 6;592(7856):756–62. doi: 10.1038/s41586-020-03039-0 (PMC8081666; doi:10.1038/s41586-020-03039-0)
Supplement: Supplementary file 1 — Details on the sample collection and analytical methods used in this study. Also includes Supplementary Results with the detailed analyses results, as well as Supplementary Tables 1, 2, 6, 7, 9, 13-16, 18, 21, 24, 25, 34, 38, 41-45 and 50. [file 41586_2020_3039_MOESM1_ESM.pdf]

nature research
https://doi.org/10.1038/s41586-020-03039-0

Supplementary information

Platypus and echidna genomes reveal mammalian biology and evolution

In the format provided by the authors and unedited

Supplementary Information

Supplementary Method

DNA, RNA extraction and sequencing

We extracted high molecular weight DNA (HMW DNA) from skeletal muscle tissue of a male platypus (*Ornithorhynchus anatinus*) Pmale09 (sourced from a New South Wales (NSW) population) and generated 101 SMRT cells using P6-C4 chemistry and RS II system (Pacific Biosciences). A total of 134 Gb (67-fold) of subread bases with an N50 read length of 19,907bp were generated.

Ultra-high molecular weight DNA (uHMW DNA) were extracted from 40mg of skeletal muscle tissue using the agarose plug BioNano Genomics protocol for Animal Tissue DNA Isolation Fibrous Tissue (#30071B) from the same platypus male individual. uHMW DNA quality was assessed by a Pulsed Field Gel assay and quantified with a Qubit 2 Fluorometer. These DNA was labeled for BioNano Genomics optical mapping using the BioNano Prep Direct Label and Stain (DLS) Protocol (30206E) and ran on Saphyr instrument chip with 1 flowcell.

We used the same unfragmented uHMW DNA to generate a linked-reads library on the 10X Genomics Chromium (Genome Library Kit & Gel Bead Kit v2 PN-120258, Genome HT Library Kit & Gel Bead Kit v2 PN-120261, Genome Chip Kit v2 PN-120257, i7 Multiplex Kit PN-120262). We sequenced this 10X library on an Illumina Novaseq S4 and generated 1,235,555,754 PE150 reads pairs.

Liver tissues were used for Hi-C library preparation. Two kinds of Hi-C libraries were built for platypus. One individual Pmale08 (sourced from a New South Wales (NSW) population) was used for Dovetail Chicago Hi-C library preparation, and we further conducted a Phase Genomics Hi-C library with Pmale09, which is the same individual in PacBio sequencing, 10X and BioNano. Pmale08 Hi-C data was generated using a Dovetail Genomics Chicago Hi-C Kit as ref1 with restriction enzyme DpnII. Sequencing was performed on an Illumina HiSeq2500, generating 222,187,634 PE100 read pairs. Platypus Pmale09 Hi-C data was generated using a Phase Genomics (Seattle, WA) Proximo Hi-C Animal Kit, which is a commercially available version of the Hi-C protocol[2]. Following the manufacturer's instructions for the kit, intact cells from two samples were crosslinked using a formaldehyde solution, digested using the Sau3AI restriction enzyme, and proximity ligated with biotinylated nucleotides to create chimeric molecules composed of fragments from different regions of the genome that were physically proximal in vivo, but not necessarily genomically proximal. Continuing with the manufacturer's protocol, molecules were pulled down with streptavidin beads and processed into an Illumina-compatible sequencing library. Sequencing was performed on an Illumina HiSeq4000, generating a total of 352,262,548 PE150 read pairs.

In terms of echidna, we extracted sufficient DNA sample from heart muscle of a male echidna (*Tachyglossus aculeatus)* Emale01 (provided by San Diego Zoo Global, sourced from Melbourne Zoo). A total of 16 libraries with insert sizes ranging from 170bp to 20kb were constructed. We performed paired-end sequencing (HiSeq 2000 platform) following the manufacturer's protocol and produced 292 Gb raw data (Supplementary Table 1). uHMW DNA samples for BioNano and 10X library was extracted from muscle tissues of a different individual Emale12 (sourced from a NSW population), and prepared in the same protocol as platypus, except that two flowcells were used in BioNano run and 1,758,748,940 PE150 10X reads pairs were sequenced. Phase Genomics Hi-C was prepared with liver tissue in the same protocol as platypus and 210,955,194 PE100 read pairs were sequenced on Illumina HiSeq4000 platform. Echidna Echidna RNA was extracted from brain, cerebellum, kidney, liver, testis and ovary. RNA-seq data were generated using a procedure described in ref3 and sequenced SE100 reads with Illumina HiSeq2500.

Bacterial Artificial Clones (BACs) were purchased from CUGI BAC/EST resource centre (Clemson, SC, USA) and the Children's Hospital Oakland Research Institute (CHORI, CA, USA) and stored in glycerol stocks at -80°C. To culture BAC clones a sterilized pipette tip was used to streak bacteria onto 1% LB Agar plates. Plates were supplemented with 12.5µg/mL chloramphenicol. Following an overnight incubation at 37°C, single colonies were selected from streak plates and placed in a solution of 40mL LB Lennox broth and 12.5µg/mL chloramphenicol to incubate overnight at 37°C whilst shaking. Broth cultures were then centrifuged (4000rpm for 15 minutes at 4°C) and pellets were dried before DNA extraction. Pellets obtained from BAC Probe Culture were resuspended in Lysis Buffer (50mM TrisPhosphate, 100mM EDTA, 100mM NaCl, 1% SDS). BACs were subjected to 10mg/mL Proteinase K incubation overnight at 60°C at 800rpm and 10mg/mL RNase incubation at 37°C for 2 hours followed by standard phenol chlorophorm extraction (A 1:1 ratio of Phenol/Chloroform:sample volume, mixed and centrifuged (14000rpm for 15 minutes at 4°C). Upper (aqueous) phase was transferred to a new 2mL tube and Phenol/Chloroform was again added, mixed by inversion and centrifuged (14000rpm for 15 minutes at 4°C). Upper (aqueous) phase was transferred to a new 1.5mL tube and 3X the sample volume of absolute ethanol was added, mixed by inversion and centrifuged (14000rpm for 15 minutes at 4°C). Without disturbing the pellet, ethanol was removed, tubes re-spun (14000rpm for 15 minutes at 4°C) and excess ethanol removed once again. Samples were dried at 37°C for 10 minutes, resuspended in MQ water and incubated at 50°C for 10 minutes to resolubilise. BAC working stocks were stored at -20°C. Extracted DNA was sequenced with Pacbio Sequel platform.

Genome assembling and curation

Platypus assembly was generated with the Vertebrate Genomes Project (VGP) v1.0 assembly pipeline. Contigs were generated from PacBio subreads using FALCON-Unzip with one round of Arrow polishing using the DNAnexus FALCON 5.1.1 and FALCON-Unzip 1.0.2 pipelines[4]. This resulted in an initial set of primary contigs, which represent a pseudo

haplotype, and a secondary set of haplotigs containing the alternative form of heterozygous alleles. The primary contig set was then iteratively scaffolded first with 10x Genomics linked reads using Scaff10X (v2.1 git 4.28.2018), then BioNano optical maps using BioNano Solve[5] (v3.2.1_04122018), and finally Dovetail Genomics Hi-C Illumina reads using SALSA[6] v2.0. An additional rounds of consensus polishing was conducted with Arrow in smrtanalysis (v5.1.0.26412) were conducted by mapping all PacBio reads. The estimated base level QV was 32.7 in Phred scale. Additional two rounds of Illumina polishing were performed using and 10X Chromium reads by mapping to both the primary and alternative scaffold sets. The final round of PacBio Arrow polishing was conducted using smrtanalysis (v5.1.0.26412), and the 10X Genomics reads were aligned with Longranger[7] (v2.2.2) before calling erroneous bases (variants) with FreeBayes[8] (v1.2.0) and correction with BCFtools[9] (v1.8). Resulting base pair QV of the combined primary and alternate assembly improved to 39.5 and to 42.4 after each round of Illumina polishing. Base error was measured from variant calls of the aligned bases. Assignment of alternative alleles in the primary assembly was further evaluated by Purge Haplotigs bitbucket 7.10.2018 to reduce artifactual duplication during curation[10]. Note that the Hi-C reads used for scaffolding were obtained from a different individual (Pmale08) because the Hi-C reads from the same individual (Pmale09) were not available at the time of assembly. Later, Hi-C reads were re-generated for Pmale09 with Phase Genomics, and scaffold breaking and joining was manual performed during curation. All scripts used in the VGP assembly pipeline are publicly available from https://github.com/VGP/vgp-assembly.

For platypus manual assembly curation, all available sequencing and mapping and previous assembly data was compared to the automatically produced assembly in gEVAL[11]. Alignment discordances were manually assessed and resolved by breaking and rejoining scaffolds. A Hi-C 2D map generated by Phase Genomics and visualized in Juicebox[12] allowed for further scaffold correction and super scaffolding in order to bring the assembly to chromosome scale. Hi-C-led changes were verified against other data in gEVAL.

For echidna genome assembling, we first filtered out low-quality and duplicated reads, and performed additional filtering using the following criteria: we excluded the reads from short-insert libraries (insert size = 170, 500 and 800 bp) with ‘N’s over 5% of the length or having more than 40 bases with the quality lower than 7, and the reads from large insert libraries (2 to 20 kb) with ‘N’s over 20% of the length or having more than 30 bases with the quality lower than 7. We also trimmed reads to exclude some low-quality regions. Finally, 189.48 Gb (76X coverage) reads (Supplementary Table 1) were retained for genome assembly using Platanus[13] (v1.2.1). The echidna genome was iteratively scaffolded first with 10x Genomics linked reads using Scaff10X v2.1 git 4.28.2018 (https://github.com/wtsi-hpag/Scaff10X), then BioNano optical maps using BioNano Solve v3.2.104122018, and finally Phase Genomics Hi-C Illumina reads using SALSA v2.0. Manual curation was also performed when anchoring scaffolds to chrX (details below).

Parameter estimation of sex-linked sequences

Parameters evaluation for X-linked sequences:

1. Since there were available anchored autosomes and chrX sequences in OANA5, we first confirmed each of them by plotting F/M depth ratio along the sequence, and classified those sequences into autosomes, PAR and X-linked. During this step, we found the F/M depth ratio of OANA5 chr14 showed a X-linked instead of autosome pattern, thus it was excluded in later parameter evaluation. For echidna we performed

homology search using protein from confirmed autosome/sex chromosomes from platypus as markers to identify autosome/sex chromosomes sequences in echidna, utilizing homology relationship between the two monotreme[14].
2. For those confirmed autosome and sex chromosome sequences, we calculated F/M depth ratio for each 5kb-non overlapping window
3. By ranging F/M depth ratio from 1.0 to 3.0 with step size 0.1, we assigned window into X-linked sequences or autosomes/PAR, and based on their origin, calculated true-positive rate, false-positive rate and false-discovery rate for autosomes, PAR and X-linked sequences, respectively.

Parameters evaluation for Y-linked sequences:
Since the published platypus assembly OANA5 is generated from a female, we instead used reads of human and mouse to perform a similar evaluation procedure as X-linked sequences, and evaluate the ratio from 0.0 to 1.0 with step size 0.1. All parameter evaluation procedures were the same except for the following parts:
1. We calculated both F/M depth ratio and F/M coverage ratio of each window
2. We used the whole window region instead of the region that were covered by both male and female as Y-linked sequences are supposed to have little female coverage.

Telomere and centromere identification

We searched for telomere monomer "TTAGGG" and "CCCTAA" in mOrnAna1, then counted the coverage of these monomers in 1kb windows by BEDTools (v2.26.0)[15]. We identified putative monotremes centromere seed sequences from Illumina data based on the same method as ref16, then searched for the sequence in mOrnAna1 by Repeatmasker. We defined a 1kb centromere or telomere region if the putative centromeric repeat or telomere monomers could cover > 70% of the region. Centromeric genome fraction in platypus and echidna was estimated with the same strategy as in ref16.

Imprinting cluster annotation

11 imprinted clusters were collected from ref17 and the protein sequences were obtained from Uniprot (http://www.uniprot.org/). Protein sequences were aligned to OANA and mOrnAna1 by TBLASTN at E-value cutoff 1e-5, respectively. Then we used Genewise to align the homologous genomic sequence with the matched protein for accurate splicing alignment. In order to verify whether the homology-annotation was reliable, we performed a BLASTP (v2.2.26) at E-value cutoff 1e-5 to human proteins set from Ensembl (release 87) for confirmation, requiring the annotated result's best hit was the query.

Immune gene annotation

Major Histocompatibility Complex (MHC) cluster annotation

First we generated pairwise synteny maps between human and each other species including mouse, opossum, koala, platypus, echidna, common wall lizard, chicken and frog. Then we extracted potential MHC region in the other species by taking the most accurate and well annotated MHC cluster in human as reference. Synteny regions were further extended 1kb at both directions, and genes within the extended regions were then BLASTP (E-value cutoff

1e-5) to whole human gene set and classified according to their best hit in human protein set. Gene classified according to the best hit in human and ref18. Manual curation was further performed to platypus and echidna Class-I and Class-II candidates. MHC phylogenetic analyses were performed with full-length coding sequences and MEGA X with maximum likelihood using four discrete categories for the Gamma distribution and 200 bootstraps. In visualization, genes within the clusters but were not classified to each class were excluded. There would be a slash when over 10 genes that didn't fall into any class existed between two classified genes; and there would be a double-slash when its flanking genes located on two different scaffolds/chromosomes.

Cathelicidin

First we obtained six frame transition of genome (human, opossum, chicken, echidna, platypus) with EMBOSS[19] (v6.5.7) and used all these translations to search for cathelicidin HMM profile (PF00666) from Pfam[20] with HMMsearch[21] (v3.1b2). Only regions with HMMer hit E-value less than 10 hits were kept. We extracted the sequences by extending 5kb flanking sequences from genome which contained cathelicidin HMM profile hit and predicted gene structure within the region by genscan[22] (v1.0). Predicted proteins were searched with HMMsearch (E-value <10) using the same PF00666 HMM profile again to make sure it contained the domain.

Chemokines

We used protein from gene set of human, opossum and chicken from Ensembl (release 87) and the two monotreme to search the chemokines profile HMM from Pfam (PF00048) with HMMsearch (v3.1b2). Only results with HMMer hits E-value <10 were considered as chemokine genes. In case that we might miss gene annotation in echidna due to the relatively poor assembly quality, we double checked the region in synteny with platypus. We extracted the region annotated genes within the region with genscan, then confirmed annotated results by searching PF00048 profile with HMMsearch (E-value<10).

Defensins

We annotated defensin in monotremes genomes with published platypus DEFA, DEFB, OavDLP, and human defensin with Genewise. Phylogenetic tree of OavDLP and DEFB-VL were constructed by RaxML with parameter set "-f a -m PROTGAMMALGX -p 12345 -x 12345 -# 100 -o -o oaDEFB-VL,taDEFB-VL" and visualized with iTol (https://itol.embl.de/).

TCRs

Published TCR constant and variable sequences from platypus, echidna, marsupials and eutherian mammals (Supplementary Table 37) were used as queries in BLASTN and TBLASTN searches of the platypus and echidna genome and predicted open reading frames, with an E-value cutoff 1e-5 to ensure no variable sequences were excluded. BLASTP searchers using the same query sequences were also performed against translated coding sequence predictions for platypus and echidna, with an E-value cutoff 1e-5. In addition, recombination signal sequences (RSS) from koala and Tasmanian devil, as well as a limited number of previously published RSS for platypus, were used to construct HMM. HMM were used as input to the program hmmer to search the platypus and echidna genome with E-value cutoff 1e-5. Top BLAST and hmmer hits were extracted from the genome using BEDTools,

and multiple sequence alignments constructed in BioEdit to identify domains and conserved amino acid residues. ClustalW[23] (v2.1) was used to align sequences in BioEdit, which was used to construct neighbour-joining phylogenetic trees in MEGA7[24] using the p-distance method, pairwise deletion and 1000 bootstrap replicates.

Tooth related gene analysis

Platypus and echidna gene set were used to search for *MMP20*, *ODAM*, *FDCSP*, *AMTN*, *AMBN*, *ENAM*, *DSPP* and *AMELX*. Protein sequences of candidate genes were BLASTP to NCBI database for confirmation. For those genes that could not be found in gene set, protein sequence obtained from Uniprot were used as query and annotated in platypus and echidna orthologous regions by exonerate[25] (v2.2.0).

Digestive genes analysis

Genes related to digestive systems like *NGN3*, *PGA*, *PGC*, *GAST*, *CTSE*, *ATP4A* and *ATP4B* were collected from ref26. Protein sequences were first TBLASTN to genomes with E-value cutoff 1e-5 and candidate gene regions were refined by GeneWise for more accurate splicing sites and gene models. We at last confirmed the gene by BLASTP it to NCBI NR database. Potential pseudogenes were defined when there's frameshift signal predicted in the gene. We confirmed the loss of these genes in a two-step procedure: first by genome synteny from LASTZ, then by mapping reads to human genome where target genes were present. To produce synteny plot for each candidate loss gene, we took three flanking genes at each side and tried annotated them with the potential loss gene. We further confirmed genomic sequence alignment within the genes' 50kb flanking region from LASTZ to check if there's exons' alignment. At last, we confirmed the loss by mapping Illumina reads to human genome. Platypus reads were from ERS2196499 and ERS2196504, and echidna reads were from all short insert-size libraries produced in this study and SRR924360. Reads mapping was done by BWA MEM with default parameters, and only primary alignment of each read was kept. Depth information was extracted and converted into BIGWIG for visualization using BEDTools and UCSC Genome Browser's utilities (http://genomewiki.ucsc.edu/index.php/) and visualized the gene region by pyGenomeTracks[27] (v2.1) to check if there's enough reads mapped to the exons of the gene. GADPH region was also plot as a control.

To validate the expression of neurogenenin-3 (*NGN3*), RNA was extracted from platypus and echidna tissues using TRIzol reagent (Ambion) and RT-reactions carried out using the SuperScript III First-Strand kit (Invitrogen) and treated with DNase. *NGN3* expression was assessed by RT-PCR using the following primers for platypus: CAAGAGCGAGCTGGTCCTGAG and CTCGATCTTGGTGAGCTTGGC and echidna: CAGCAAACAGAGGCGGAACC and AGCGTCTCGATCTTGGTGAG and control β-actin primers: GCCCATCTACGAAGGTTACGC and AAGGTCGTTTCGTGGATACCAC.

Chemosensory receptor gene analysis

The annotation method of each gene family is described below. For phylogenetic analysis, multiple alignments of annotated sequences were constructed using E-INS-i in MAFFT[28] (v6.857b) and MUSCLE[29] (v3.8.31). The phylogenetic trees were constructed by RAxML (v8.2.12) and TreeBeST[30] (v1.9.2) and visualized by MEGA (v5.5.2) and ggtree[31] (v1.16.6).

Bitter taste receptor genes (*TAS2R*s)

TAS1R and *TAS2R* genes are annotated from the platypus and echidna genome assemblies according to the same method used in previous study[32] based on the BLAST and TOPCONS[33] (v2.0). The best-scoring maximum-likelihood tree of mammalian-wide *TAS2R* genes was inferred using RAxML under the GTR + Γ model with 1000 bootstrap replicates based on the multiple alignment of amino acid sequences constructed by E-INS-i in MAFFT. This is the amino-acid-based unrooted tree of intact, truncated and pseudogenized *TAS2R*s that have 250 amino acids and more. Amino acid sequences of pseudogenes were deduced after their disrupted sites were excluded based on multiple alignment of nucleotide sequences of phylogenetically-close intact *TAS2R*s. These gene set consists of *TAS2R*s from human and mouse (eutherians[34]), koala, wallaby, Tasmanian devil and opossum (marsupials[32]) and platypus and echidna (monotremes) and additionally rabbit *TAS2R301* and *TAS2R302* and aye-aye *TAS2R372*, whose orthologs were not possessed by human and mouse. Gene trees were visualized using MEGA.

Olfactory receptor genes (*OR*s)

First, we extracted whole amino acid sequences of all chordate intact *OR*s previously reported by ref35 as queries. Redundant query sequences were integrated with the 70% identity threshold as representatives by CD-HIT[36] (v4.6.8). Next, tblastn-fast[37]/exonerate-based search using the FATE[38] (v2.7.0) platform were performed to platypus and echidna assemblies. The UniRef50 protein database with GHOSTZ[39] (v1.0.2) were searched to filter out sequences that did not have any hits with *OR*-related keywords in the top 10. Finally, using the hit sequences, following procedures are repeated for 20 times. Repeated and redundant intact sequences were masked by RepeatMasker v4.0.9 with RepBase-20181026 library and then by CD-HIT (95% identity threshold). Using remaining intact sequences, FATE search was performed based on the dc-megablast[37] and exonerate, and the obtained sequences were filtered as described above.

For the final dataset, multiple alignment by MUSCLE and topology prediction by TOPCONS in amino acid sequences assigned as "intact" genes by FATE examined the presence of adequate seven-transmembrane domains of GPCR to ascertain whether the genes are correctly intact or preorganized genes. Moreover, based on pairwise amino acid sequence similarities, intact *OR* genes are classified into class I or non-class I genes[35].

Vomeronasal receptor genes (*V1R*s)

The annotation of *V1R* genes in the new platypus and echidna assemblies was performed in the completely same way of their *OR* genes described above, using all intact mammalian *V1R*s previously reported by ref40 as the first extracted queries.

Hemoglobin degradation genes analysis

Protein sequences of haptoglobin (*HP*) and its flanking genes (*IST1, PKD1L3, DHODH, HPR, TXNL4B, DHX38, PMFBP1 and DPEP1*) were obtained from Uniprot, and annotated in genomes of human, opossum, platypus (mOrnAna1), echidna, chicken, emu, anole lizard, turtle, frog and fugu in the same way as digestive system related gene part, and confirm monotremes' *HP* loss in the same way. To construct the gene tree of *HP* and its related proteases family, we obtained members from CAFE's result in this study, aligned their protein sequences by PRANK with default parameters, and built gene phylogenetic trees

based on protein sequences by RaxML with parameter set “-f a -m PROTGAMMALGX -p 12345 -x 12345 -# 100” and visualized with ggtree.

PIT54 protein sequence was obtained from Uniprot and the homologous annotation in monotremes was done in the same way as above. We also obtained monotreme scavenger receptor cysteine-rich (SRCR) group B family members using protein queries from NCBI and annotated them in a similar pipeline as HP. For SRCR SR-I subgroup members we additionally required that annotated protein aligning rate > 75% and a 20kb maximum intron length. Annotation result was further curated by inspecting the alignment between platypus and echidna from LASTZ. SRCR group B members in other species were obtained from Ensembl (release 87). Reads depth distribution of platypus chr3 and echidna scaffold_17 in a non-overlapping 500bp was extracted to confirm SCART1 gene number difference between monotremes. In phylogenetic tree construction, protein sequences were aligned by MUSCLE (v3.8.31) with default parameters and fed to TreeBeST with parameter set “nj -t mm -b 100” and visualized online at iTOL (https://itol.embl.de/).

Reproductive gene analysis

Sequences of human, cow, chicken, opossum, koala, platypus and echidna were obtained from NCBI (Supplementary Table 52). For annotation of the genes of interest in the monotreme genomes, published sequences of platypus and echidna were used if available, otherwise sequences of human, cow, koala, and grey short-tailed opossum were used as queries in TBLASTN searches of the platypus and echidna genomes, with an E-value cutoff 1e-5 to ensure no variable sequences were excluded. Confirmation of identity was performed via BLASTN and BLASTP searches and subsequent syntenic analyses.

Monotreme syntenic regions of interest surrounding these genes were then manually identified and synteny maps constructed between the monotremes and other species including human, cow, opossum, koala and chicken as relevant, based on data retrieved from the NCBI gene database. If the monotreme genes of interest were not identified via TBLASTN, syntenic regions of the genomes were extracted and annotated with genscan and additional manual annotations performed if required based on previously published sequences. Annotated results were confirmed by Clustal Omega (v1.2.2) alignments with other species based on conserved exon-exon structure and amino acid residues.

Supplementary Results

Monotreme distribution

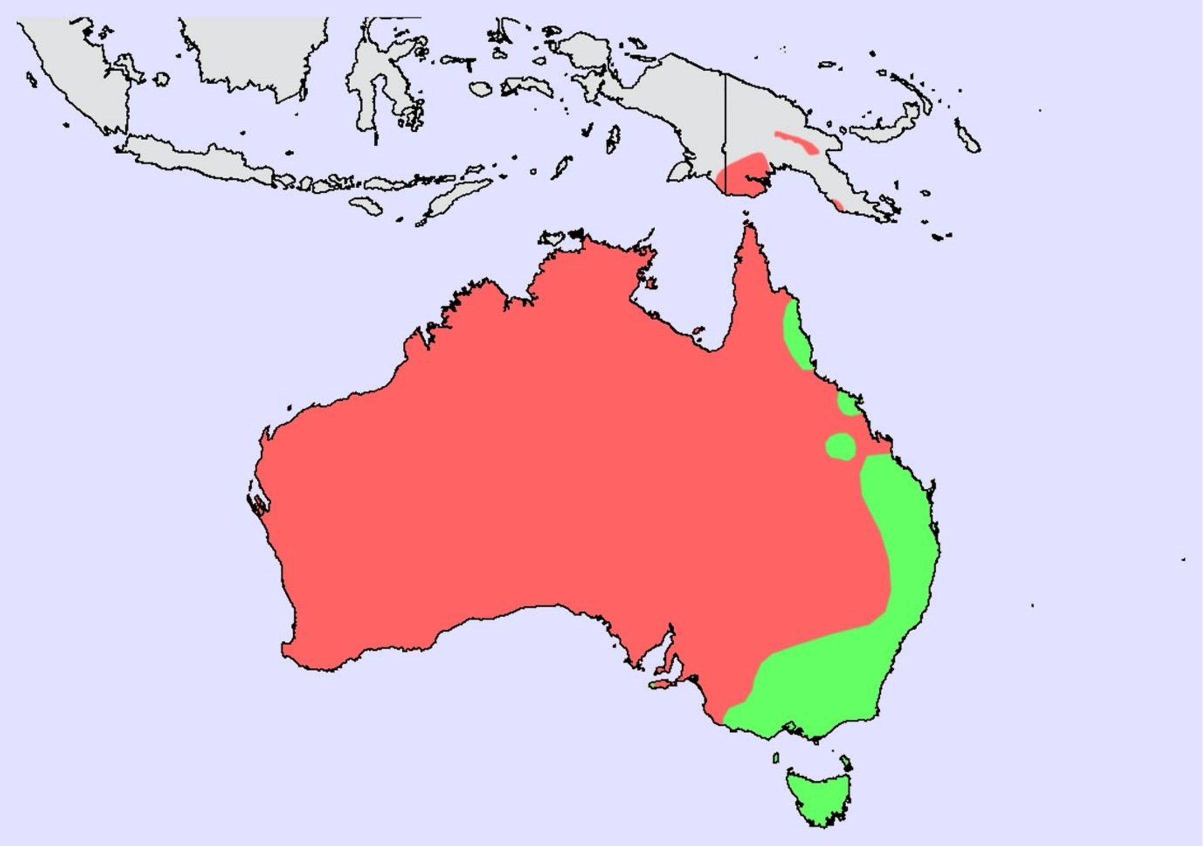

Supplementary Figure 1. Distribution map of platypus (*Ornithorhynchus anatinus)* and short-beaked echidna (*Tachyglossus aculeatus)* in Australasia. Images courtesy Professor Geoff Shaw, University of Melbourne. Green: platypus (overlaps with echidna); red and green: short-beaked echidna. Echidna data is obtained from IUCN red list https://www.iucnredlist.org/species/41312/21964662 and platypus data is obtained from the Australian platypus conservancy https://platypus.asn.au/distribution-numbers/.

Telomere and centromere in monotremes

The new platypus assembly has reach chromosome-scale with the help of various kinds of sequencing technologies and the available BAC and gene markers. In addition to the improvement on the sequence continuity and gene annotation, the assembly also harbors the complex repetitive regions like telomeres and centromeres. Thirteen telomeric regions were identified in mOrnAna1 (Supplementary Table 8). Besides, we also identified centromere monomers in the two monotremes. Centromere monomer's characters in platypus and echidna are different in the aspect of monomers' length, GC content and the estimated genome fraction (platypus 0.2669, echidna 0.7383). Moreover, monomer's pairwise BLASTN did not produce any hit under Evalue cutoff 10. This suggested that monotremes' centromeres are not conserved and are fast-evolving.

Centromere monomer sequence in monotremes

```
>platypus
AGGTCAGGCCGGGAGCAGAGGTTTAGCCGGGAGAAAAGGTCAGGCCGGGAGCA
A
>echidna
ATCATTATTATCATTTAAAATGGGGATAAAACTGTGAGCCCCCCGTGGGACAACC
TGATCACCTTGTAACCTCCCCAGCGCTTAGAACAGTGCTTTGCACATAGTAAGCG
CTTAATAAATGCCATCATTATTATTATTATTATT
```

Genome architecture comparison between monotremes and therians

Both monotremes have smaller genome size at around 2Gb than that of many other terrestrial mammals (at around 3Gb) (Supplementary Table 13). The percentage of repeat elements in both species was at a similar level to that in other mammals (Supplementary Table 12). Nevertheless, from the comparison of the repetitive elements in these representative mammals we found that the total LINE content is also at a similar level between monotremes and therians. The major difference comes from the subtypes of each repeat family: LINE/L2 is dominant in both platypus and echidna, while LINE/L1 is dominant in human, mouse and opossum.

BUSCO analysis of the new monotreme assemblies

We performed BUSCO (v3.0.2) analysis to the two monotreme assemblies in genome mode with database "mammalia_odb9". The result suggested that mOrnAna1 has a higher genome completeness in gene level compared to OANA5, and even achieves a similar level to human and mouse (Supplementary Table 14). On the other hand, the completeness of mTacAcu1 is lower than mOrnAna1, possibly because of the high heterozygosity rate of the sequenced individual (~0.465%) and the assembly is based on Illumina reads.

Improved assemblies increase the power and resolution of cross-species comparison

With the new platypus assembly, we detected 15,447 one-to-one orthologs between platypus (mOrnAna1) and human (hg38), a 31% increase from OANA5 (Supplementary Table 16). The whole genome synteny analysis between human and platypus led to the identification of synteny blocks spanning 81.11% of human genome. The total syntenic length is 1.4-fold longer than detected with the previous version and the syntenic block N50 size increased three-fold (Supplementary Table 16). For example, the improved synteny length allowed us to retrieve 10 of the 11 therian imprinting gene clusters in the platypus genome, compared to only four clusters detected in OANA5 (Extended Data Fig. 2b, Supplementary Table 17). The echidna-human one-to-one orthologs number is at a similar level with that between OANA5 and human while the synteny block N50 achieves a 2.2-fold increase (Supplementary Table 16).

Noncoding regions contribution to mammalian evolution

Non-coding genomic regions can have significant contribution in shaping phenotypic evolution by regulating gene expression[41]. By performing whole genome comparison across vertebrate clades, we identified 14.08Mb Mammal Specific Highly Conserved Elements (MSHCEs). These genomic regions are highly conserved across mammals but are evolving under neutrality in other vertebrates. While 89.85 % of MSHCEs are located in non-coding regions (Extended Data Fig. 2c) and 32.75% overlapped with known regulatory elements (i.e. promoters, enhancers, CTCF binding sites (Supplementary Table 18)), indicating they might function as *cis*-regulatory elements of their nested genes. We defined gene as MSHCE-associated if there are MSHCEs within the genic and flanking regions (10kb) (Supplementary Table 19). The top 300 genes are enriched not only in general RNA metabolism and expression regulation, but also in several development processes. Interestingly, we found enriched GO terms like "neuron differentiation" (FDR-adjusted $P = 5.93e^{-10}$, chi-square test), "regulation of nervous system development" (FDR-adjusted $P = 8.14e^{-8}$, chi-square test) and "nervous system development" (false discovery rate (FDR)-adjusted $P = 1.03e^{-8}$, chi-square test) (Extended Data Fig. 2d, Supplementary Table 20), suggesting MSHCEs might have contributed to brain development in mammals. For example, we found an 876bp MSHCE located in an intron of *BCL11A*. This gene is expressed in the cortical plate and intermediate zone during neocortex development, and controls cell polarity as well as radial migration to the upper layer[42]. *BLC11A* also participates in neuron subtype development and the lack of *BCL11A* during development would cause hypoplasia of the upper cortical layer[43]. This MSHCE seems to function as an enhancer of *BLC11A* during neocortex development as it overlaps a H3K27ac ChIP-seq signal was found overlapping with the element in both 8.5 postconception weeks (p.c.w) and 12 p.c.w when human brain is developing in embryo[44] (Extended Data Fig. 2e). Many other genes which are known to participate in neurogenesis and neocortex development like *NFIB*[45] and *MEF2C*[46] have also been found to be MSHCE-associated, suggesting that these highly conserved non-coding sequences in the mammalian lineage might have contributed to the development of the neocortex, which is a defining trait for the mammalian brain, though further experimental validation is required to elucidate the role of these elements in gene expression regulation.

Evaluation of ancestral karyotype reconstruction

The marsupial and therian ancestral karyotypes have been reconstructed before[47]. Therefore, it offers an opportunity for us to compare the results from the two independent efforts. The therian ancestral karyotypes in our new reconstructions is overall consistent with Deakin's construction (Supplementary Fig. 2, Supplementary Table 21). This suggests that our independent effort to order and orientate CAR into ancestral chromosomes based on gene synteny information is reliable. For example, the marsupial ancestral chromosome 6 is homologous to human chromosomes in the order of 8, 4 and 11, and the therian ancestral chromosome 2 is homologous to human chromosomes in the order of 3, 21, X, 2, 13, 11, 3, 2, 3. The major difference of the two reconstructions comes from the number of ancestral therian chromosomes: there are 2n=36 ancestral chromosomes in our reconstruction while

there are 2n=38 ancestral chromosomes in Deakin's reconstruction. In our version, the major orthologous sequence to human chr19 is derived from a single therian ancestral chromosome 17 while it was separated into two therian ancestral chromosomes in Deakin's result. The presence of this ancestral chromosome 17 was supported by the well conserved synteny of the entire human chr19, opossum chr3, Tasmanian devil chr1 and platypus chrX1, thus confirming our accurate construction.

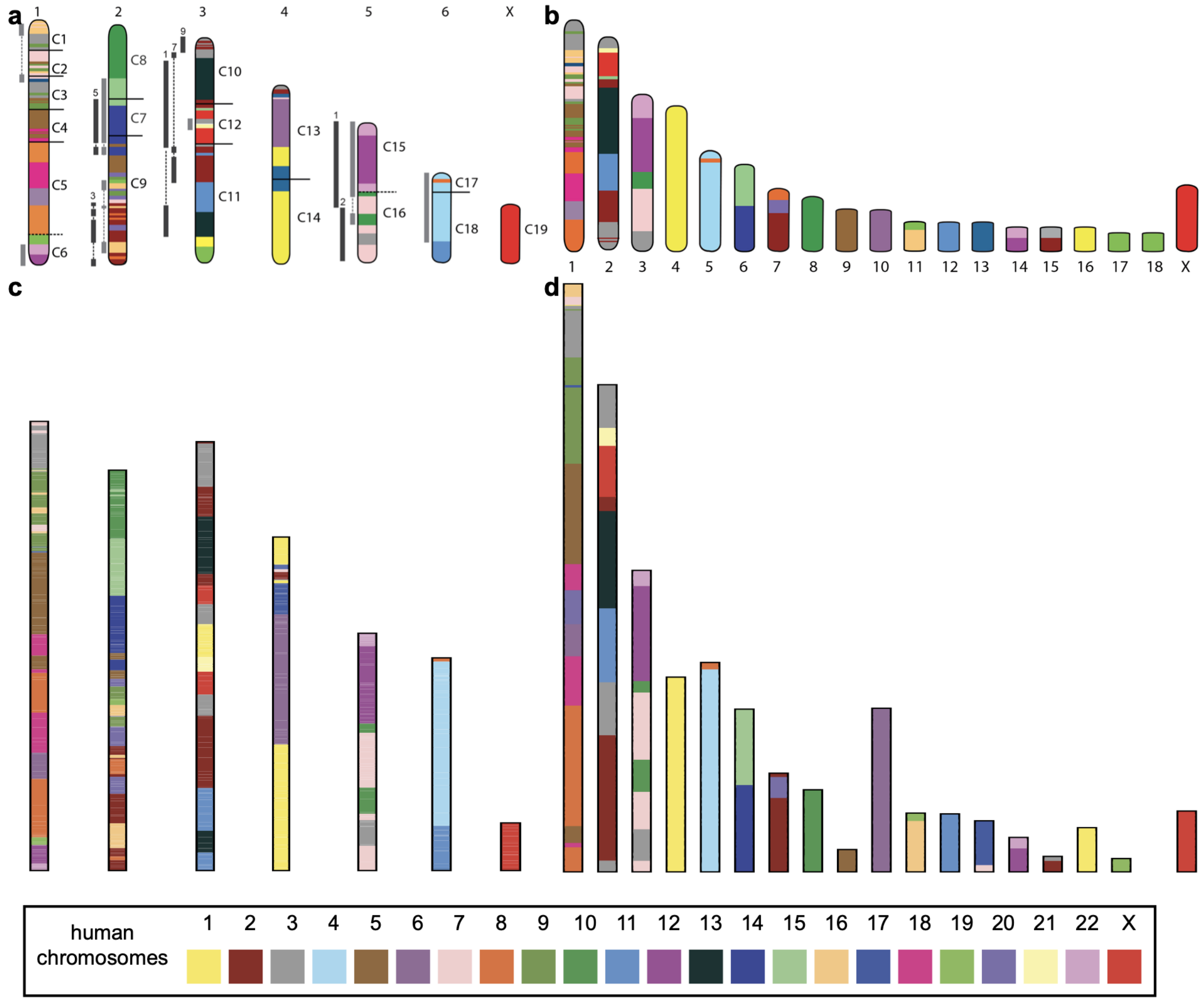

Supplementary Figure 2. Comparison of karyotype reconstruction between Deakin's and our version. The upper panel shows Deakin's reconstruction for marsupial ancestral karyotype (**a**) and therian ancestral karyotype (**b**) adapted from Figure 9, Deakin et al. *BMC Evol Biol*. 2013. **c** and **d** show our reconstruction version for marsupial and therian ancestral karyotype.

Apart from being largely consistent between the two reconstruction results, we further integrate some of the Deakin's results to improve our final version of reconstruction. For example, the reconstruction by Deakin et al. further helps to associate CAR10,35 to therian ancestral chromosome 7, CAR20,30 to therian ancestral chromosome 13 and CAR24,25,26,5,28 along with 7 and others to therian ancestral chromosome 1.

One of the most important parameters is the resolution of the conserved block, i.e. the minimal size of conserved synteny blocks used in reconstruction. Generally, the smaller the

minimum size of conserved block is, the finer resolution of genome rearrangement results that the reconstruction can provide. But that would also result in a more fragmented reconstruction. To test if our reconstruction can be changed with different resolution, we also performed the analysis in 300kb resolution. This resulted in reconstructions with the exact same reconstruction (marsupial ancestor karyotype 2n=14, therian ancestor karyotype 2n=36 and mammalian ancestor karyotype 2n=60 under both 500kb and 300kb). All the above evidence suggests our reconstruction result is reliable and robust.

Rearrangement during karyotype evolution

With the rearrangement events in each lineage, we found that the marsupial lineage experienced a higher number of genomic rearrangements (N=249 for opossum and N=191 for Tasmanian devil) than platypus and human lineages (N=156 and 165, respectively; Fig. 1b, Supplementary Table 28), and the number of fissions and fusions from mammalian ancestor to therian ancestors is higher than average. Nonetheless, when accounting for the branch length, the rearrangement rates across the included species are not significantly different from each other (FDR corrected p-value > 0.3, Supplementary Table 28). As repetitive sequences are usually associated with evolutionary breakpoint regions (EBRs)[48], the higher repeat content of marsupial genomes (~52% compared to ~45% in eutherians) could suggest a higher propensity of these species to chromosomal rearrangements.

Evolution of immune gene families in monotremes

The major histocompatibility complex (MHC) is a gene-dense region found in all jawed vertebrate genomes containing a large number of genes involved in immune pathways. The classical class I and class II MHC genes, in particular, play central roles in host self/non-self recognition and immune defense against infections and cancers. These genes are known to be subject to strong selection pressures posed by constantly evolving pathogens and in turn play an important part in driving adaptive evolution in vertebrates. The highly contiguous genome assemblies of the platypus and echidna have helped reveal several unique features of the monotreme MHC. In both genomes, MHC genes form two separate gene clusters located on two different sex chromosomes (Extended Data Fig. 3a, Supplementary Table 31), which is consistent with previous observations[49]. The majority of the genes reside within PAR regions, though several are found in SDR. Interestingly, among the genes located in the SDR, we identified a class II alpha-like gene in both species (Extended Data Fig. 3a) that lacks an exon encoding the peptide-binding domain of class II molecules, but showed moderate transcription level in the testis in both species (Supplementary Table 32). There is a possibility that this gene may have evolved from an MHC class II gene, then diverged to play a specific function in male monotremes. Based on the gene expression profile and sequence similarity with previously reported monotreme MHC transcripts, we identified three putatively classical class I (class Ia) genes in the platypus (*Oran-MHC-I-1*, *-2*, and *-3*) and one in the echidna (*Taac-MHC-I-1*; Supplementary Table 33). These genes are co-localized with antigen-processing genes (*TAP1*, *TAP2*, *PSMB8*, *PSMB9*) and class II genes, which is a feature more commonly seen in non-mammalian lineages. Unexpectedly, a strong orthologous relationship was detected between platypus and echidna class Ia genes (Extended

Data Fig. 3b). MHC class Ia genes are known to evolve rapidly with short gene turnover time[50], which has resulted in a lack of orthology between genes from distantly related species in most lineages studied so far (e.g. marsupial genes in Extended Data Fig. 3b) and different from the phylogeny of class II genes (Extended Data Fig. 3c, d. Considering that the two monotremes diverged ~55 million years ago and underwent distinct adaptive evolution, it is surprising for their class Ia genes to exhibit such high levels of similarity. This may be an indication of selection pressure by pathogens that are unique to monotremes leading to a high degree of conservation in monotreme MHC genes.

The current version of the platypus genome has also greatly improved annotation of T cell receptors (*TCR*s). We found that monotreme TCR constant (C) and variable (V) sequences are similar to those in marsupials[32,51] in terms of gene content and organization (Supplementary Table 34-36). However, Vγ sequences have expanded in monotremes relative to therian mammals[52,53] (Extended Data Fig. 3e). As VJ recombination is important for variation of the CDR (antigen binding region) this expansion may increase the CDR variability which may be beneficial for monotremes in adapting to viral threats. However, a previous study[53] suggests that the platypus may preferentially express Vγ sequences in circulating γδ T lymphocytes, and some genomic Vγ sequences may not be utilized.

The genome also enabled annotation of antimicrobial peptide genes. Defensins are a family of antimicrobial peptides which detect bacteria, viruses, fungi and parasites[54]. In platypus, the defensin genes gave rise to unique venom defensin-like peptides which are the major components of platypus venom[55]. Our new platypus genome assembly places the three venom defensin-like genes (*OavDLP-A*, *-B* and *-C*) within a beta defensin gene cluster (Extended Data Fig. 3f, Supplementary Table 38). In echidna, however, only a single *OavDLP* was identified and we predict this is a pseudogene as only one exon was remained (Extended Data Fig. 3g). The echidna pseudo-*OavDLP* appears most similar to platypus *OavDLP-A* (Extended Data Fig. 3g, h) and as this is predicted to be the last of the *OavDLP*s to have appeared[55] would suggest that the ancestral monotreme had all three venom defensin genes and that echidna has lost the other two (i.e. *OavDLP-B* and *-C*). Echidna crural gland transcriptome analysis did not detect OavDLPs[56] and it appears that the echidna's capacity to deliver venom decayed with the gland taking on a new functional role as the new species adapted to life on land.

Analysis of genes related to digestive system

Monotremes have an extremely small stomach and have lost almost all of their stomach glands[57]. Genes involved in gastric acid secretion (*GAST*, *ATP4A* and *ATP4B*) and protein degradation (*PGA*, *PGC* and *CTSE*) are also lost in monotremes (Extended Data Fig. 8b-f, Supplementary Table. 43). This suggests that this loss of gastric genes occurred in the monotreme ancestor more than 55 MYA. However, contrary to previous reports[26], *NGN3*, a key gene for stomach and pancreatic islet development, is present and expressed in both monotremes (Extended Data Fig. 8g).

Chemosensory receptor analysis

Bitter taste receptor genes (*TAS2R*s)

Mammalian taste receptors are expressed in taste buds of the oral cavity and encoded by 2 GPCR (G protein-coupled receptor) gene families, *TAS1R*s and *TAS2R*s. The *TAS1R* family in mammals generally consist of *TAS1R1*, *TAS1R2* and *TAS1R3*. The TAS1R1 and TAS1R3 heterodimer is responsible for the umami taste receptor, whereas TAS1R2 and TAS1R3 heterodimer is for the sweet taste receptor. In contrast, gene numbers of the *TAS2R* family drastically vary among mammalian species because TAS2Rs recognize the broad range of numerous harmful compounds as bitter taste receptors. The platypus assembly has completely intact *TAS1R1*, *-2* and *-3*. The echidna assembly has truncated (i.e., not completely sequenced) exons of *TAS1R*, *-2* and *-3* and there are no sequences causing nonsense or frameshift mutations. Therefore, extant monotremes and the last common ancestor (LCA) of them have presumably functional umami and sweet taste receptors.

The platypus and echidna assemblies have 7 and 3 intact genes and 1 and 5 pseudogenes of *TAS2R*s, respectively (Supplementary Table 45). There were no truncated genes. These are comparatively smaller sized repertoire than general terrestrial therian mammals[34]. Previous studies detected 28 *TAS2R* orthologous gene groups (OGGs) in eutherians and 27 *TAS2R* OGGs in marsupials[32,34,58]. These OGG numbers are corresponding to the numbers of *TAS2R*s that the eutherian or marsupial LCA have.

The topology is substantially the same to previous studies[32,34,58-60]. The known 28 eutherian and 27 marsupial OGGs are distinctly confirmed. As new findings, the number of monotreme OGGs is only 7, defined here as *TAS2R801* and *TAS2R802* (scattered in the tree) and *TAS2R810-814* (assembled in a cluster, marked in Fig. 4a). The 7 monotreme OGGs are presumably intact in the LCA of platypus and echidna. After split of these species, platypus gained a duplicate of *TAS2R813*, pseudogenized *TAS2R814*, and thus retains 7 *TAS2R*s. On the other hand, echidna gained a duplicate of *TAS2R812* but both were pseudogenized independently, and moreover, *TAS2R801*, *TAS2R810* and *TAS2R811* were pseudogenized. Finally, echidna has only 3 *TAS2Rs*.

TAS2R801 is clustered with therian *TAS2R1* (bootstrap probability = 98%), whereas *TAS2R802* is clustered with eutherian *TAS2R5* and *-6* and marsupial *TAS2R701* (bootstrap probability = 70%). These may be orthologous relationships, respectively. The monotreme cluster, *TAS2R810-814* is the sister group of the therian cluster I, suggesting that the LCA of mammals have an ancestral gene of these paralogs and that independent massive gene expansions occurred as the therian cluster I and monotreme cluster after split these two mammalian groups. Interestingly, the therian cluster I includes *TAS2R16* and *TAS2R41* (*Tas2r126* in mouse nomenclature), which were activated by highly toxic cyanogenic glycosides (β-D-glucopyranosides) at the cellular level in human and mouse, respectively. Toxic eucalyptus leaf-eating koala also has many *TAS2R* duplicates in this cluster[32]. Although the diet of the common ancestor of monotremes is not clear, the therian cluster I and monotreme cluster probably encode the important toxic sensing taste receptors.

When the viewpoint is changed from monotremes to therians, therians have 2 additional clusters. The therian cluster II consists of 2 eutherian (*TAS2R39* and *TAS2R40*) and 2 marsupial (*TAS2R702* and *TAS2R703*) genes. The therian cluster III is the supercluster that includes the 2 eutherian (I and II) and 3 marsupial (I, II and III) clusters. Unless drastic

expansion and/or contraction events occurred in ancestral monotremes of the uninvestigable geological span of the Mesozoic era (before split between platypus and echidna) (Extended Data Fig. 9a), the therian cluster III most especially characterizes the specificity of therian taste evolution.

Olfactory receptor genes (*OR*s)

Platypus and echidna have 299 and 693 intact, 2 and 6,537 truncated and 259 and 8,268 pseudogenized *OR* genes, respectively (Fig. 4a, Supplementary Table 46). Platypus *OR* genes are scattered on 13 of the 21 autosomes, 4 of the 5 X and 1 of the 5 Y chromosomes. Echidna *OR* genes are also widely scattered on 10,636 of 1,340,799 scaffolds. The numbers of truncated and pseudogenized *OR*s in echidna are seemingly extraordinary and the prominently largest among previously reported chordate species[35]. Intact, truncated and pseudogenized *OR*s in echidna are not localized in certain scaffolds (186, 6443 and 4291, respectively), and therefore, echidna genome has experienced frequent *OR* duplication and pseudogenization events.

The numbers of intact class I *OR* genes in platypus and echidna are 48 (16% of all the *OR* genes) and 100 (14%), respectively (Supplementary Table 46). These ratios are not deviated from terrestrial therians (generally 10-20%). All the 48 intact class I *OR* genes in platypus is exclusively clustered in the chromosome 2, that is, there is no non-class I *OR* genes in the platypus chromosome 2, spanning over 1.2 Mb (Extended Data Fig. 9b, Supplementary Table 46). In human (eutherian) and opossum (marsupial), class I *OR* genes are clustered in the chromosome 11 and 4, respectively, but the chromosome of each species has also other *OR* genes[61,62]. A previous study found that the nucleotide sequence of an enhancer element for mouse class I *OR* genes, the J element is conserved through mammalian species[63]. We confirmed that the platypus J element is located at the 22-kb downstream of an end of the *OR* gene cluster (Extended Data Fig. 9b).

Vomeronasal 1 receptor genes (*V1R*s)

Platypus and echidna have 262 and 28 intact, 6 and 671 truncated and 624 and 710 pseudogenized *V1R* genes, respectively (Fig. 4a, Supplementary Table 47). Platypus *V1R* genes are scattered on 10 of the 21 autosomes, 4 of the 5 X and 1 of the 5 Y chromosomes. Echidna *V1R* genes are also widely scattered on 1052 of the 1,340,799 scaffolds. We confirmed platypus has the largest *V1R* repertoire among vertebrates, but this is the platypus-specific event because echidna has the small *V1R* repertoire. There are three monotreme-specific clusters (Fig. 4a). This is also consistent with a previous platypus study[40].

Supplementary Tables

Supplementary Table 1. Statistics of echidna Illumina data.

Insert Size (bp)	#Library	Raw Reads Length (bp)	Total Data (Gb)	Filtered Reads Length (bp)	Filtered Data (Gb)	Sequence Depth (X)	Physical Depth (X)
170	4	100	83.77	95	69.96	27.98	25.04
500	3	100	53.77	95	43.17	17.27	45.44
800	2	100	32.20	95	24.73	9.89	41.66
2,000	7	49	45.48	49	26.32	10.53	214.83
5,000	3	49	30.15	49	10.63	4.25	217.01
10,000	2	49	20.45	49	11.33	4.53	462.48
20,000	3	49	26.64	49	3.34	1.34	272.84
Total	24		292.46		189.48	75.79	1279.3

Supplementary Table 2. Assembly metrics comparison. "mOrnAna1, t4p" and "mTacAc1, s3" are the assemblies before curation and anchoring. "mOrnAna1" and "mTacAcu1" are the anchored assemblies that used in further analysis.

	platypus (OANA5)	platypus (mOrnAna1, t4p)	platypus (mOrnAna1)	echidna (mTacAcu1, s3)	echidna (mTacAcu1)
total length (bp)	2,073,117,877	1,879,296,647	1,877,718,804	2,311,984,458	2,311,986,058
#contigs	535,990	1,229	1,233	1,509,654	1,509,655
total contig length (bp)	1,917,665,773	1,862,555,161	1,862,555,161	2,124,847,162	2,124,847,162
maximum contig length (bp)	245,967	68,365,665	68,365,665	369,388	369,388
contig N50 (bp)	10,808	15,022,425	15,022,425	20,562	20,562
#scaffolds	291,092	732	693	1,340,809	1,340,799
maximum scaffold length (bp)	59,581,953	194,022,312	186,506,016	13,746,727	130,997,925
scaffold N50 (bp)	833,983	41,107,209	83,338,043	31,932,450	32,506,442
total gap length (bp)	155,446,883	16,741,486	15,163,643	187,137,296	187,138,896
gap ratio (%)	7.50	0.89	0.81	8.09	8.09

Supplementary Table 6. X-linked and Y-linked sequence anchoring percentage.

chr		mOrnAna1			mTacAcu1		
		length (bp)	percentage (%)	#gene	length (bp)	percentage (%)	#gene
X	X1	80,248,076	42.78	827	82,564,875	44.56	764
	X2	14,285,706	7.62	233	16,861,564	9.10	231
	X3	13,764,197	7.34	79	16,295,257	8.79	128
	X4	3,635,000	1.94	63	60,946,805	32.89	354
	X5	60,821,577	32.42	438	1,082,127	0.58	25
	Xun	14,834,039	7.91	529	7,552,524	4.08	233
	total	187,588,595	100.00	2,169	185,303,152	100.00	1,735
Y	Y1	1,234,000	8.68	8			
	Y2	5,167,112	36.34	25			
	Y3	2,861,818	20.13	17			
	Y4	1,018,000	7.16	15			
	Y5	2,789,633	19.62	39			
	Yun	1,147,005	8.07	9	8,634,534	100.00	100
	total	14,217,568	100.00	113	8,634,534	100.00	100

Supplementary Table 7. Platypus anchoring percentage comparison.

chr	expected length (Mb)	OANA5		mOrnAna1	
		length (bp)	percentage (%)	length (bp)	percentage (%)
1	188.00	60,877,203	32.38	186,506,016	99.21
2	192.00	55,155,618	28.73	168,695,607	87.86
3	152.00	59,595,685	39.21	138,511,391	91.13
4	136.00	58,995,228	43.38	135,051,410	99.30
5	108.00	24,616,169	22.79	110,293,030	102.12
6	226.00	37,524,201	16.60	51,493,492	22.78
7	80.00	40,039,088	50.05	83,338,043	104.17
8	66.00	10,039,024	15.21	70,949,814	107.50
9	58.00	1,915,287	3.30	61,861,223	106.66
10	56.00	11,243,762	20.08	59,444,696	106.15
11	60.00	6,809,224	11.35	63,506,031	105.84
12	58.00	15,872,666	27.37	60,456,049	104.23
13	50.00	0	0.00	42,294,642	84.59
14	50.00	3,880,500	7.76	52,889,406	105.78
15	48.00	20,740,701	43.21	26,375,019	54.95
16	44.00	13,568,040	30.84	46,397,275	105.45
17	42.00	2,790,086	6.64	45,536,670	108.42
18	38.00	15,946,317	41.96	44,684,501	117.59
19	36.00	12,745,677	35.40	35,120,541	97.56
20	30.00	4,815,888	16.05	30,535,534	101.79
21	24.00	2,508,583	10.45	24,514,859	102.15
X1	118.00	52,648,064	44.62	125,040,914	105.97
X2	46.00	14,240,315	30.96	29,662,106	64.48
X3	46.00	22,729,282	49.41	33,863,336	73.62
X4	20.00	665,755	3.33	8,639,456	43.20
X5	66.00	31,058,238	47.06	70,139,320	106.27
sum	2,038.00	581,020,601	28.51	1,805,800,381	88.61
total		2,073,148,626	28.03	1,879,304,658	96.09

Supplementary Table 9. Summary statistics of OANA5 gap-filling.

type	#gap	gap size in OANA5 (bp)	gap size percentage (%)
BadMapped	17,325	8,320,982	5.87
Filled	174,307	127,212,185	89.69
Overlapped	41,264	5,163,223	3.64
Unmapped	1,554	1,133,248	0.80
total	234,450	141,829,638	100.00

Supplementary Table 13. Genomic features in representative therians and monotremes.

species	genome size (bp)	repeat content (%)	gene						
			#gene	average gene length (bp)	average mRNA length (bp)	#exon per gene	average exon length (bp)	average intron length (bp)	average intergenic length (bp)
human	3,099,750,718	53.84	20,423	49,552	1,691	9.72	174	5,486	104,932
mouse	2,730,871,774	45.09	22,621	36,979	1,581	8.85	179	4,511	84,519
opossum	3,605,631,728	55.87	21,327	51,157	1,584	8.78	180	6,368	126,850
platypus	1,877,718,804	50.41	20,742	38,183	1,647	9.38	176	4,361	62,382
echidna	2,311,986,258	48.01	22,029	30,741	1,231	7.13	173	4,811	70,091

Supplementary Table 14. Summary statistics of BUSCO analysis of different representative mammals.

assembly	Complete (%)		Fragmented (%)	Missing (%)
	Single-copy (%)	Duplicated (%)		
platypus (mOrnAna1)	93.3	1.0	2.8	2.9
platypus (OANA5)	70.3	0.4	18.6	10.7
echidna (mTacAcu1)	71.0	0.5	11.0	17.5
opossum	88.7	1.0	4.2	6.1
mouse	94.1	1.2	2.3	2.4
human	94.1	0.8	2.5	2.6

Supplementary Table 15. Substitution rate of representative mammals.

species	substitution rate (substitution per site per MY)
Ornithorhynchus anatinus	2.59E-03
Tachyglossus aculeatus	2.66E-03
Monodelphis domestica	2.25E-03
Loxodonta africana	2.52E-03
Dasypus novemcinctus	2.56E-03
Sorex araneus	3.74E-03
Bos taurus	2.87E-03
Canis familiaris	2.74E-03
Myotis lucifugus	2.88E-03
Mus musculus	3.67E-03
Tupaia belangeri	3.02E-03
Macaca mulatta	2.57E-03
Homo sapiens	2.53E-03

Supplementary Table 16. Orthologues and synteny statistics between species and human. Synteny block, coverage are calculated based on human genome.

species	#one-to-one orthologue	#synteny block	synteny blocks N50 (bp)	synteny block total length (bp)	synteny blocks coverage (%)
mouse	17,144	813	39,657,131	2,865,053,216	92.43
opossum	15,024	1,468	26,710,328	2,658,401,169	85.76
platypus (OANA5)	11,780	5,450	5,078,526	1,856,044,738	59.88
platypus (mOrnAna1)	15,447	1,474	17,331,489	2,514,077,806	81.11
echidna	11,538	2,232	11,405,632	2,259,513,958	72.89
chicken	13,013	1,785	14,640,912	2,374,030,124	76.59

Supplementary Table 18. MSHCE length percentage that overlap with regulatory elements from Ensembl regulatory build.

regions	% MSHCE
CTCF binding site	3.80
TF binding site	0.47
enhancer	6.55
open chromatin	2.85
promoter	6.24
promoter flanking	14.76

Supplementary Table 21. CAR joining evidence by synteny is highly consistent with Deakin's reconstruction result.

Reconstruction node	CAR joining evidence by synteny (CAR ID)	Supported by Deakin's reconstruction?
Marsupialia	6,4	Yes
Theria	11, 18,31	Yes
	13, 17	Yes
	2,14,19	Yes
	7,21,23,34,36	Yes
	3,6,32	Yes

Supplementary Table 24. Length of human sequences used in ancestral karyotype reconstruction.

chromosome	total length (bp)	500k resolution		300k resolution	
		length (bp)	percentage (%)	length (bp)	percentage (%)
chr1	248,956,422	181,388,338	72.86	180,286,599	72.42
chr2	242,193,529	174,808,008	72.18	174,394,156	72.01
chr3	198,295,559	146,023,224	73.64	147,724,490	74.50
chr4	190,214,555	157,927,496	83.03	138,117,544	72.61
chr5	181,538,259	106,090,834	58.44	106,112,797	58.45
chr6	170,805,979	145,709,119	85.31	145,951,162	85.45
chr7	159,345,973	97,000,819	60.87	100,250,888	62.91
chr8	145,138,636	113,834,608	78.43	103,963,446	71.63
chr9	138,394,717	82,338,286	59.50	78,985,933	57.07
chr10	133,797,422	95,199,054	71.15	97,824,703	73.11
chr11	135,086,622	100,076,611	74.08	99,797,173	73.88
chr12	133,275,309	88,294,842	66.25	97,511,041	73.17
chr13	114,364,328	76,168,076	66.60	72,309,693	63.23
chr14	107,043,718	65,361,594	61.06	68,414,600	63.91
chr15	101,991,189	58,821,878	57.67	52,258,087	51.24
chr16	90,338,345	46,885,156	51.90	53,736,088	59.48
chr17	83,257,441	35,953,036	43.18	45,483,468	54.63
chr18	80,373,285	62,384,266	77.62	62,167,108	77.35
chr19	58,617,616	13,018,864	22.21	10,825,671	18.47
chr20	64,444,167	42,930,136	66.62	44,077,286	68.40
chr21	46,709,983	14,084,535	30.15	14,948,813	32.00
chr22	50,818,468	17,819,313	35.06	19,577,222	38.52
chrX	156,040,895	84,457,369	54.13	81,322,084	52.12
total	3,031,042,417	2,006,575,462	66.20	1,996,040,052	65.85

Supplementary Table 25. Length of platypus sequences used in ancestral karyotype reconstruction.

chromosome	total length (bp)	500k resolution		300k resolution	
		length (bp)	percentage (%)	length (bp)	percentage (%)
chr1	186,506,016	138,619,108	74.32	135,254,525	72.52
chr2	171,719,663	117,492,679	68.42	125,815,787	73.27
chr3	141,987,082	86,413,956	60.86	87,461,729	61.60
chr4	136,094,389	104,750,328	76.97	103,173,691	75.81
chr5	110,293,030	70,321,713	63.76	67,686,590	61.37
chr6	51,493,492	20,715,301	40.23	22,037,823	42.80
chr7	83,338,043	57,383,944	68.86	59,852,364	71.82
chr8	70,949,814	57,964,096	81.70	56,599,638	79.77
chr9	61,861,223	51,094,439	82.60	54,495,824	88.09
chr10	59,444,696	35,997,765	60.56	33,782,136	56.83
chr11	63,506,031	49,462,411	77.89	49,667,045	78.21
chr12	60,456,049	50,242,609	83.11	49,173,495	81.34
chr13	42,294,642	33,396,815	78.96	35,795,232	84.63
chr14	52,889,406	42,240,114	79.86	43,586,662	82.41
chr15	26,375,019	17,653,914	66.93	16,540,514	62.71
chr16	46,397,275	37,350,324	80.50	36,086,236	77.78
chr17	45,536,670	21,845,623	47.97	23,075,652	50.67
chr18	44,684,501	30,737,101	68.79	29,636,161	66.32
chr19	35,120,541	31,178,457	88.78	31,470,521	89.61
chr20	30,535,534	23,347,077	76.46	22,059,922	72.24
chr21	24,514,859	7,983,421	32.57	12,059,852	49.19
chrX1	125,040,914	55,509,869	44.39	54,053,715	43.23
chrX2	29,662,106	16,354,898	55.14	17,580,259	59.27
chrX3	33,863,336	19,338,228	57.11	19,252,680	56.85
chrX4	8,639,456	1,222,765	14.15	2,922,329	33.83
chrX5	70,139,320	45,568,242	64.97	43,083,168	61.43
total	1,813,343,107	1,224,185,197	67.51	1,232,203,550	67.95

Supplementary Table 34. Number of T cell receptor variable (V) and constant (C) sequences identified in human, mouse, gray short-tailed opossum, koala, echidna and platypus (α, β, δ, γ and μ). The number of sequences with complete open reading frames are shown in brackets.

TCR	human[66]	mouse[66]	opossum[51]	koala[32]	echidna	platypus
TRA/DV	62 (47)	114 (78)	94 (60)	52 (52)	85 (78)	116 (103)[65]
TRAC	1 (1)	1 (1)	1 (1)	1 (1)	1 (1)[64]	1 (1)
TRDC	1 (1)	1 (1)	1 (1)	1 (1)	1 (1)	1 (1)
TRBV	64-67 (40-48)	35 (21-22)	36 (27)	33 (33)	47 (34)	45 (27)
TRBC	2 (2)	2 (2)	4 (4)	2 (2)	2 (2)[64]	2 (2)
TRGV	12-15 (4-6)	7 (7)	9 (9)	4 (4)	49 (39)	56 (51)
TRGC	2 (2)	4 (3)	1 (1)	1 (1)	4 (4)	4 (4)[53]
TRMV	N/A	N/A	6 (6)	2 (2)	6 (6)	19 (14)[52]
TRMVj	N/A	N/A	8 (8)	3 (3)	0	0
TRMC	N/A	N/A	8 (8)	3 (3)	2 (2)	15 (11)[52]

Supplementary Table 38. Immune gene families size in monotremes.

	defensins		cathelicidins	chemokines
	alpha	beta		
platypus	4	6 *DefB-VL* + 3 *OavDLP*	10	31
echidna	4	5 *DefB-VL*	6	23

Supplementary Table 41. Interaction count of interphase nuclei in platypus fibroblasts.

		# cells with interaction	# cells without interaction	total # cells
Y2-Y3	rep1	11	174	185
	rep2	22	184	206
	rep3	23	235	258
Y2-X1	rep1	1	257	258
	rep2	7	243	250
	rep3	4	201	205
Y2-WSB1	rep1	6	292	298
	rep2	2	260	262
	rep3	2	218	220

Supplementary Table 42. Genomic location of teeth-related genes in monotremes.

gene	platypus			echidna		
	chr	start	end	chr	start	end
MMP20	NA	NA	NA	NA	NA	NA
ODAM	NA	NA	NA	NA	NA	NA
FDCSP	NA	NA	NA	NA	NA	NA
AMTN	NA	NA	NA	NA	NA	NA
AMBN	chr10	6,785,229	6,813,579	NA	NA	NA
ENAM	chr10	6,827,221	6,842,117	NA	NA	NA
DSPP	chr12	54,432,549	54,451,115	scaffold_34	6,847,499	6,866,850
AMELX	chr15	21,553,288	21,556,174	scaffold_36	5,331,550	5,331,963

Supplementary Table 43. Potential digestive related gene loss check in both monotremes. Y: present; F: fragmented; M: missing; P: potential pseudogene; alnrate = annotated_protein_length/query_protein_length.

	platypus				echidna				conclusion
	annotation	synteny	mapping	conclusion	annotation	synteny	mapping	conclusion	
NGN3	Y	-	-	Y	Y	-	-	Y	present in both monotremes
PGA	M	M	M	M	F (alnrate= 0.20)	F	M	F	loss in platypus & fragmented in echidna
PGC	M	M	M	M	M	M	M	M	loss in monotreme
GAST	M	M	M	M	M	M	M	M	loss in monotreme?
CTSE	F (alnrate= 0.07)	M	M	M	F (alnrate= 0.06)	M	M	M	loss in monotreme
ATP4A	F (alnrate= 0.12)	F	-	M	F (alnrate= 0.04)	F	-	M	loss in monotreme
ATP4B	P & F (alnrate= 0.60)	-	-	P	F (alnrate= 0.23)	F	-	F?	do not function in monotreme?

Supplementary Table 44. Number of chemosensory receptor genes. Numbers and percentages in parentheses are for class I ORgenes.

		# Intact	# Truncated	# Pseudogenized
TAS2R	Human[34]	26	NA	10
	Opossum[32]	27	4	5
	Platypus	7	0	1
	Echidna	3	0	5
OR	Human[62]	398 (59; 15%)	0	442
	Opossum[61]	1188 (221; 19%)	10	294
	Platypus	299 (48; 16%)	2	259
	Echidna	693 (100; 14%)	6537	8268
V1R	Human[38]	2	0	163
	Opossum[40]	95	35	
	Platypus	262	6	624
	Echidna	28	671	710

Supplementary Table 45. Annotation of bitter taste receptor genes (*TAS2R*s).

Species	Gene	Chromosome	Strand	Start	End	State
Platypus	*TAS2R801*	chr13	+	37,537,979	37,538,908	intact
Platypus	*TAS2R814P*	chr13	-	37,660,283	37,661,126	pseudo
Platypus	*TAS2R813B*	chr13	-	37,661,984	37,662,910	intact
Platypus	*TAS2R812*	chr13	-	37,663,987	37,664,898	intact
Platypus	*TAS2R813A*	chr13	-	37,666,009	37,666,935	intact
Platypus	*TAS2R811*	chr13	-	37,668,077	37,669,000	intact
Platypus	*TAS2R810*	chr13	-	37,671,841	37,672,764	intact
Platypus	*TAS2R802*	chr2	-	171,365,488	171,366,450	intact
Echidna	*TAS2R801P*	scaffold_31	+	15,796,290	15,797,194	pseudo
Echidna	*TAS2R814*	scaffold_31	-	15,934,053	15,934,979	intact
Echidna	*TAS2R812BP*	scaffold_31	-	15,936,415	15,937,216	pseudo
Echidna	*TAS2R813*	scaffold_31	-	15,938,137	15,939,066	intact
Echidna	*TAS2R812AP*	scaffold_31	-	15,940,157	15,941,106	pseudo
Echidna	*TAS2R811P*	scaffold_31	-	15,942,561	15,943,595	pseudo
Echidna	*TAS2R810P*	scaffold_31	-	15,944,976	15,945,854	pseudo
Echidna	*TAS2R802*	scaffold_83	+	198,588	199,553	intact

Supplementary Table 50. Genomic location of identified SRCR family groupB members in platypus and echidna.

species	gene ID	gene name	scaffold ID	strand	start	end
platypus	oaDMBT1	*DMBT1*	chr16	+	18,650,512	18,727,280
platypus	oaSCART1a	*SCART1*	chr3	-	131,497,831	131,516,237
platypus	oaSCART1b	*SCART1*	chr3	-	131,531,345	131,553,956
platypus	oaSCART1c	*SCART1*	chr3	-	131,571,293	131,597,494
platypus	oaSCART1d	*SCART1*	chr3	-	131,616,545	131,642,454
platypus	oaSCART1e	*SCART1*	chr3	-	131,661,673	131,688,768
platypus	oaSCART1f	*SCART1*	chr3	-	131,708,176	131,732,546
platypus	oaSCART1g	*SCART1*	chr3	-	131,746,713	131,773,387
platypus	oaSCART1h	*SCART1*	chr3	-	131,803,576	131,818,423
platypus	oaWC1.1a	*WC1*	chr16	-	45,124,928	45,157,896
platypus	oaWC1.1b	*WC1*	chr16	-	45,213,165	45,249,816
echidna	taDMBT1	*DMBT1*	scaffold_17	-	22,476,768	22,534,675
echidna	taSCART1a	*SCART1*	scaffold_209	+	314,022	340,897
echidna	taSCART1b	*SCART1*	scaffold_209	+	358,189	384,178
echidna	taWC1.1a	*WC1*	scaffold_258	-	272,970	334,081
echidna	taWC1.1b	*WC1*	scaffold_258	-	299,995	357,652
platypus	oaCD5	*CD5*	chr3	-	17,606,919	17,634,788
platypus	oaCD5L	*CD5L*	chrX5	+	5,003,278	5,005,714
platypus	oaCD6	*CD6*	chr3	-	17,691,893	17,751,613
platypus	oaSSC4D	*SSC4D*	chr17	+	2,073,904	2,085,046
platypus	oaSSC5D	*SSC5D*	chr10	-	52,255,089	52,268,384
echidna	taCD5	*CD5*	scaffold_18	+	37,088,019	37,107,097
echidna	taCD6	*CD6*	scaffold_18	+	36,962,031	36,970,455
echidna	taSSC5D	*SSC5D*	scaffold_626	+	21,924	33,445

Reference

1 Putnam, N. H. *et al.* Chromosome-scale shotgun assembly using an in vitro method for long-range linkage. *Genome Res* **26**, 342-350, doi:10.1101/gr.193474.115 (2016).

2 Lieberman-Aiden, E. *et al.* Comprehensive mapping of long-range interactions reveals folding principles of the human genome. *Science* **326**, 289-293, doi:10.1126/science.1181369 (2009).

3 Cardoso-Moreira, M. *et al.* Gene expression across mammalian organ development. *Nature* **571**, 505-509, doi:10.1038/s41586-019-1338-5 (2019).

4 Chin, C. S. *et al.* Phased diploid genome assembly with single-molecule real-time sequencing. *Nat Methods* **13**, 1050-1054, doi:10.1038/nmeth.4035 (2016).

5 Lam, E. T. *et al.* Genome mapping on nanochannel arrays for structural variation analysis and sequence assembly. *Nat Biotechnol* **30**, 771-776, doi:10.1038/nbt.2303 (2012).

6 Ghurye, J. *et al.* Integrating Hi-C links with assembly graphs for chromosome-scale assembly. (Bioinformatics, 2018).

7 Bishara, A. *et al.* Read clouds uncover variation in complex regions of the human genome. *Genome Res* **25**, 1570-1580, doi:10.1101/gr.191189.115 (2015).

8 Garrison, E. & Marth, G. Haplotype-based variant detection from short-read sequencing. *arXiv e-prints* (2012). <https://ui.adsabs.harvard.edu/abs/2012arXiv1207.3907G>.

9 Li, H. A statistical framework for SNP calling, mutation discovery, association mapping and population genetical parameter estimation from sequencing data. *Bioinformatics* **27**, 2987-2993, doi:10.1093/bioinformatics/btr509 (2011).

10 Roach, M. J., Schmidt, S. A. & Borneman, A. R. Purge Haplotigs: allelic contig reassignment for third-gen diploid genome assemblies. *BMC Bioinformatics* **19**, 460, doi:10.1186/s12859-018-2485-7 (2018).

11 Chow, W. *et al.* gEVAL - a web-based browser for evaluating genome assemblies. *Bioinformatics* **32**, 2508-2510, doi:10.1093/bioinformatics/btw159 (2016).

12 Durand, N. C. *et al.* Juicebox Provides a Visualization System for Hi-C Contact Maps with Unlimited Zoom. *Cell Syst* **3**, 99-101, doi:10.1016/j.cels.2015.07.012 (2016).

13 Kajitani, R. *et al.* Efficient de novo assembly of highly heterozygous genomes from whole-genome shotgun short reads. *Genome Res* **24**, 1384-1395, doi:10.1101/gr.170720.113 (2014).

14 Rens, W. *et al.* The multiple sex chromosomes of platypus and echidna are not completely identical and several share homology with the avian Z. *Genome Biol* **8**, R243, doi:10.1186/gb-2007-8-11-r243 (2007).

15 Quinlan, A. R. & Hall, I. M. BEDTools: a flexible suite of utilities for comparing genomic features. *Bioinformatics* **26**, 841-842, doi:10.1093/bioinformatics/btq033 (2010).

16 Melters, D. P. *et al.* Comparative analysis of tandem repeats from hundreds of species reveals unique insights into centromere evolution. *Genome Biol* **14**, R10, doi:10.1186/gb-2013-14-1-r10 (2013).

17 Pask, A. J. *et al.* Analysis of the platypus genome suggests a transposon origin for mammalian imprinting. *Genome Biol* **10**, R1, doi:10.1186/gb-2009-10-1-r1 (2009).

18 Belov, K. *et al.* Reconstructing an ancestral mammalian immune supercomplex from a marsupial major histocompatibility complex. *PLoS Biol* **4**, e46, doi:10.1371/journal.pbio.0040046 (2006).

19 Rice, P., Longden, I. & Bleasby, A. EMBOSS: the European Molecular Biology Open Software Suite. *Trends Genet* **16**, 276-277, doi:10.1016/s0168-9525(00)02024-2 (2000).
20 Finn, R. D. *et al.* Pfam: the protein families database. *Nucleic Acids Res* **42**, D222-230, doi:10.1093/nar/gkt1223 (2014).
21 Mistry, J., Finn, R. D., Eddy, S. R., Bateman, A. & Punta, M. Challenges in homology search: HMMER3 and convergent evolution of coiled-coil regions. *Nucleic Acids Res* **41**, e121, doi:10.1093/nar/gkt263 (2013).
22 Burge, C. & Karlin, S. Prediction of complete gene structures in human genomic DNA. *J Mol Biol* **268**, 78-94, doi:10.1006/jmbi.1997.0951 (1997).
23 Larkin, M. A. *et al.* Clustal W and Clustal X version 2.0. *Bioinformatics* **23**, 2947-2948, doi:10.1093/bioinformatics/btm404 (2007).
24 Kumar, S., Stecher, G. & Tamura, K. MEGA7: Molecular Evolutionary Genetics Analysis Version 7.0 for Bigger Datasets. *Mol Biol Evol* **33**, 1870-1874, doi:10.1093/molbev/msw054 (2016).
25 Slater, G. S. & Birney, E. Automated generation of heuristics for biological sequence comparison. *BMC Bioinformatics* **6**, 31, doi:10.1186/1471-2105-6-31 (2005).
26 Ordonez, G. R. *et al.* Loss of genes implicated in gastric function during platypus evolution. *Genome Biol* **9**, R81, doi:10.1186/gb-2008-9-5-r81 (2008).
27 Ramirez, F. *et al.* High-resolution TADs reveal DNA sequences underlying genome organization in flies. *Nat Commun* **9**, 189, doi:10.1038/s41467-017-02525-w (2018).
28 Katoh, K. & Standley, D. M. MAFFT multiple sequence alignment software version 7: improvements in performance and usability. *Mol Biol Evol* **30**, 772-780, doi:10.1093/molbev/mst010 (2013).
29 Edgar, R. C. MUSCLE: multiple sequence alignment with high accuracy and high throughput. *Nucleic Acids Res* **32**, 1792-1797, doi:10.1093/nar/gkh340 (2004).
30 Ruan, J. *et al.* TreeFam: 2008 Update. *Nucleic Acids Res* **36**, D735-740, doi:10.1093/nar/gkm1005 (2008).
31 Yu, G., Lam, T. T., Zhu, H. & Guan, Y. Two Methods for Mapping and Visualizing Associated Data on Phylogeny Using Ggtree. *Mol Biol Evol* **35**, 3041-3043, doi:10.1093/molbev/msy194 (2018).
32 Johnson, R. N. *et al.* Adaptation and conservation insights from the koala genome. *Nat Genet* **50**, 1102-1111, doi:10.1038/s41588-018-0153-5 (2018).
33 Tsirigos, K. D., Peters, C., Shu, N., Kall, L. & Elofsson, A. The TOPCONS web server for consensus prediction of membrane protein topology and signal peptides. *Nucleic Acids Res* **43**, W401-407, doi:10.1093/nar/gkv485 (2015).
34 Hayakawa, T., Suzuki-Hashido, N., Matsui, A. & Go, Y. Frequent expansions of the bitter taste receptor gene repertoire during evolution of mammals in the Euarchontoglires clade. *Mol Biol Evol* **31**, 2018-2031, doi:10.1093/molbev/msu144 (2014).
35 Niimura, Y. On the origin and evolution of vertebrate olfactory receptor genes: comparative genome analysis among 23 chordate species. *Genome Biol Evol* **1**, 34-44, doi:10.1093/gbe/evp003 (2009).
36 Li, W. & Godzik, A. Cd-hit: a fast program for clustering and comparing large sets of protein or nucleotide sequences. *Bioinformatics* **22**, 1658-1659, doi:10.1093/bioinformatics/btl158 (2006).
37 Camacho, C. *et al.* BLAST+: architecture and applications. *BMC Bioinformatics* **10**, 421, doi:10.1186/1471-2105-10-421 (2009).

38 Moriya-Ito, K., Hayakawa, T., Suzuki, H., Hagino-Yamagishi, K. & Nikaido, M. Evolution of vomeronasal receptor 1 (V1R) genes in the common marmoset (Callithrix jacchus). *Gene* **642**, 343-353, doi:10.1016/j.gene.2017.11.048 (2018).

39 Suzuki, S., Kakuta, M., Ishida, T. & Akiyama, Y. Faster sequence homology searches by clustering subsequences. *Bioinformatics* **31**, 1183-1190, doi:10.1093/bioinformatics/btu780 (2015).

40 Young, J. M., Massa, H. F., Hsu, L. & Trask, B. J. Extreme variability among mammalian V1R gene families. *Genome Res* **20**, 10-18, doi:10.1101/gr.098913.109 (2010).

41 Lindblad-Toh, K. *et al.* A high-resolution map of human evolutionary constraint using 29 mammals. *Nature* **478**, 476-482, doi:10.1038/nature10530 (2011).

42 Wiegreffe, C. *et al.* Bcl11a (Ctip1) Controls Migration of Cortical Projection Neurons through Regulation of Sema3c. *Neuron* **87**, 311-325, doi:10.1016/j.neuron.2015.06.023 (2015).

43 Woodworth, M. B. *et al.* Ctip1 Regulates the Balance between Specification of Distinct Projection Neuron Subtypes in Deep Cortical Layers. *Cell Rep* **15**, 999-1012, doi:10.1016/j.celrep.2016.03.064 (2016).

44 Reilly, S. K. *et al.* Evolutionary genomics. Evolutionary changes in promoter and enhancer activity during human corticogenesis. *Science* **347**, 1155-1159, doi:10.1126/science.1260943 (2015).

45 Piper, M. *et al.* NFIB-mediated repression of the epigenetic factor Ezh2 regulates cortical development. *J Neurosci* **34**, 2921-2930, doi:10.1523/JNEUROSCI.2319-13.2014 (2014).

46 Li, H. *et al.* Transcription factor MEF2C influences neural stem/progenitor cell differentiation and maturation in vivo. *Proc Natl Acad Sci U S A* **105**, 9397-9402, doi:10.1073/pnas.0802876105 (2008).

47 Deakin, J. E. *et al.* Reconstruction of the ancestral marsupial karyotype from comparative gene maps. *BMC Evol Biol* **13**, 258, doi:10.1186/1471-2148-13-258 (2013).

48 O'Connor, R. E. *et al.* Reconstruction of the diapsid ancestral genome permits chromosome evolution tracing in avian and non-avian dinosaurs. *Nat Commun* **9**, 1883, doi:10.1038/s41467-018-04267-9 (2018).

49 Dohm, J. C., Tsend-Ayush, E., Reinhardt, R., Grutzner, F. & Himmelbauer, H. Disruption and pseudoautosomal localization of the major histocompatibility complex in monotremes. *Genome Biol* **8**, R175, doi:10.1186/gb-2007-8-8-r175 (2007).

50 Nei, M., Gu, X. & Sitnikova, T. Evolution by the birth-and-death process in multigene families of the vertebrate immune system. *Proc Natl Acad Sci U S A* **94**, 7799-7806, doi:10.1073/pnas.94.15.7799 (1997).

51 Parra, Z. E. *et al.* Comparative genomic analysis and evolution of the T cell receptor loci in the opossum Monodelphis domestica. *BMC Genomics* **9**, 111, doi:10.1186/1471-2164-9-111 (2008).

52 Wang, X., Parra, Z. E. & Miller, R. D. Platypus TCRmu provides insight into the origins and evolution of a uniquely mammalian TCR locus. *J Immunol* **187**, 5246-5254, doi:10.4049/jimmunol.1101113 (2011).

53 Parra, Z. E., Arnold, T., Nowak, M. A., Hellman, L. & Miller, R. D. TCR gamma chain diversity in the spleen of the duckbill platypus (Ornithorhynchus anatinus). *Dev Comp Immunol* **30**, 699-710, doi:10.1016/j.dci.2005.10.002 (2006).

54 Lehrer, R. I., Lichtenstein, A. K. & Ganz, T. Defensins: antimicrobial and cytotoxic peptides of mammalian cells. *Annu Rev Immunol* **11**, 105-128, doi:10.1146/annurev.iy.11.040193.000541 (1993).

55 Whittington, C. M. *et al.* Defensins and the convergent evolution of platypus and reptile venom genes. *Genome Res* **18**, 986-994, doi:10.1101/gr.7149808 (2008).
56 Wong, E. S., Nicol, S., Warren, W. C. & Belov, K. Echidna venom gland transcriptome provides insights into the evolution of monotreme venom. *PLoS ONE* **8**, e79092, doi:10.1371/journal.pone.0079092 (2013).
57 Griffiths, M. The Biology of Monotremes.,(Academic Press Inc.: New York.). (1978).
58 Liu, Z. *et al.* Dietary specialization drives multiple independent losses and gains in the bitter taste gene repertoire of Laurasiatherian Mammals. *Front Zool* **13**, 28, doi:10.1186/s12983-016-0161-1 (2016).
59 Behrens, M., Korsching, S. I. & Meyerhof, W. Tuning properties of avian and frog bitter taste receptors dynamically fit gene repertoire sizes. *Mol Biol Evol* **31**, 3216-3227, doi:10.1093/molbev/msu254 (2014).
60 Li, D. & Zhang, J. Diet shapes the evolution of the vertebrate bitter taste receptor gene repertoire. *Mol Biol Evol* **31**, 303-309, doi:10.1093/molbev/mst219 (2014).
61 Niimura, Y. & Nei, M. Extensive gains and losses of olfactory receptor genes in mammalian evolution. *PLoS ONE* **2**, e708, doi:10.1371/journal.pone.0000708 (2007).
62 Niimura, Y., Matsui, A. & Touhara, K. Acceleration of Olfactory Receptor Gene Loss in Primate Evolution: Possible Link to Anatomical Change in Sensory Systems and Dietary Transition. *Mol Biol Evol* **35**, 1437-1450, doi:10.1093/molbev/msy042 (2018).
63 Iwata, T. *et al.* A long-range cis-regulatory element for class I odorant receptor genes. *Nat Commun* **8**, 885, doi:10.1038/s41467-017-00870-4 (2017).
64 Belov, K., Miller, R. D., Ilijeski, A., Hellman, L. & Harrison, G. A. Isolation of monotreme T-cell receptor alpha and beta chains. *Immunogenetics* **56**, 164-169, doi:10.1007/s00251-004-0679-9 (2004).
65 Parra, Z. E., Lillie, M. & Miller, R. D. A model for the evolution of the mammalian t-cell receptor alpha/delta and mu loci based on evidence from the duckbill Platypus. *Mol Biol Evol* **29**, 3205-3214, doi:10.1093/molbev/mss128 (2012).
66 Giudicelli, V., Chaume, D. & Lefranc, M. P. IMGT/GENE-DB: a comprehensive database for human and mouse immunoglobulin and T cell receptor genes. *Nucleic Acids Res* **33**, D256-261, doi:10.1093/nar/gki010 (2005).
