# Supplementary material for: Platypus and echidna genomes reveal mammalian biology and evolution
Source: Nature. 2021 Jan 6;592(7856):756–62. doi: 10.1038/s41586-020-03039-0 (PMC8081666; doi:10.1038/s41586-020-03039-0)
Supplement: Supplementary file 2 — Reporting Summary [file 41586_2020_3039_MOESM2_ESM.pdf]

nature research

Corresponding author(s): Guojie Zhang

Last updated by author(s): Jul 6, 2020

Reporting Summary

Nature Research wishes to improve the reproducibility of the work that we publish. This form provides structure for consistency and transparency in reporting. For further information on Nature Research policies, see our Editorial Policies and the Editorial Policy Checklist.

Statistics

For all statistical analyses, confirm that the following items are present in the figure legend, table legend, main text, or Methods section.

n/a	Confirmed	
☐	☒	The exact sample size (*n*) for each experimental group/condition, given as a discrete number and unit of measurement
☐	☒	A statement on whether measurements were taken from distinct samples or whether the same sample was measured repeatedly
☐	☒	The statistical test(s) used AND whether they are one- or two-sided<br>*Only common tests should be described solely by name; describe more complex techniques in the Methods section.*
☒	☐	A description of all covariates tested
☐	☒	A description of any assumptions or corrections, such as tests of normality and adjustment for multiple comparisons
☐	☒	A full description of the statistical parameters including central tendency (e.g. means) or other basic estimates (e.g. regression coefficient) AND variation (e.g. standard deviation) or associated estimates of uncertainty (e.g. confidence intervals)
☐	☒	For null hypothesis testing, the test statistic (e.g. *F*, *t*, *r*) with confidence intervals, effect sizes, degrees of freedom and *P* value noted<br>*Give P values as exact values whenever suitable.*
☐	☒	For Bayesian analysis, information on the choice of priors and Markov chain Monte Carlo settings
☒	☐	For hierarchical and complex designs, identification of the appropriate level for tests and full reporting of outcomes
☒	☐	Estimates of effect sizes (e.g. Cohen's *d*, Pearson's *r*), indicating how they were calculated

Our web collection on statistics for biologists contains articles on many of the points above.

Software and code

Policy information about availability of computer code

Data collection	Fluorescence in situ hybridization (FISH) data were collected Nikon Ti Microscope using NIS-Elements software (supplied with a Nikon Eclipse-Ti inverted epifluorescent microscope) and processed with ImageJ.
Data analysis	Common bioinformatic and statistical analysis software packages were used, including: smrtanalysis (v5.1.0.26412), FALCON (v5.1.1), FALCON-Unzip (v1.0.2), Scaff10X (v2.1 git 4.28.2018), BioNano Solve (v3.2.1_04122018), SALSA (v2.0), Longranger (v2.2.2), FreeBayes (v1.2.0), BCFtools (v1.8), gEVAL (https://vgp-geval.sanger.ac.uk), Platanus (v1.2.1), BWA (v0.7.12), Samtools (v1.2), BLAST (v2.2.26), NCBI BLAST (v2.2.31), minimap2 (v2.13), Mashmap (v2.0), RepeatMasker (v4.0.6), trf (v4.07), ProteinMasker (v4.0.6), RepeatModeler (v1.0.8), GeneWise (v2.4.1), Augustus (v3.0.3), HISAT2 (v2.0.4), stringTie (v1.2.3), Iprscan (v5.16-55.0), BEDTools (v2.26.0), LASTZ (v1.04.00), BUSCO (v3.0.2), RaxML (v8.2.4), PAML (v4.7), MULTIZ (v11.2), PHAST (v1.5), orthoMCL (v2.0.9), CAFE (v4.2), REVIGO (http://revigo.irb.hr/), inferCARs (2006-Jun-16), ANGES (v1.01), MCScanX (08-05-2012), GRIMM (v2.1), DESeq (v1.28.0), HiC-Pro (v2.10.0), ggplot2 (v3.2.1), HiCExplorer (v3.0), MEME (v4.12.0), EMBOSS (v6.5.7), hmmer (v3.1b2), genscan (v1.0), MEGA (X, 7, v5.5.2), exonerate (v2.2.0), pyGenomeTracks (v2.1), MAFFT (v6.857b), MUSCLE (v3.8.31), TreeBest (v1.9.2), ggtree (v1.16.6), TOPCONS (v2.0), CD-HIT (v4.6.8), FATE (v2.7.0), GHOSTZ (v1.0.2), Clustal Omega (v1.2.4), Clustal W (v2.1), ImageJ (v2.0.0), NIS-ELements software (v4.20.00). Custom scripts are open source and available on GitHub at https://github.com/ZhangLabSZ/MonotremeGenome.

For manuscripts utilizing custom algorithms or software that are central to the research but not yet described in published literature, software must be made available to editors and reviewers. We strongly encourage code deposition in a community repository (e.g. GitHub). See the Nature Research guidelines for submitting code & software for further information.

Data

Policy information about availability of data

All manuscripts must include a data availability statement. This statement should provide the following information, where applicable:

- Accession codes, unique identifiers, or web links for publicly available datasets
- A list of figures that have associated raw data
- A description of any restrictions on data availability

Platypus whole genome shotgun project has been deposited at GenBank under the project accession PRJNA489114 and PRJNA489115, CNSA (https://db.cngb.org/cnsa/) of CNGBdb with accession CNP0000130 and GenomeArk at https://vgp.github.io/genomeark-curated-assembly/Ornithorhynchus_anatinus/. Echidna whole genome shotgun project has been deposited at GenBank under the project accession PRJNA576333, CNSA of CNGBdb with accession CNP0000697 and GenomeArk at https://vgp.github.io/genomeark/Tachyglossus_aculeatus/. Echidna RNA-seq has been deposited at GenBank under the project accession PRJNA591380 and CNSA of CNGBdb with accession CNP0000779.

Field-specific reporting

Please select the one below that is the best fit for your research. If you are not sure, read the appropriate sections before making your selection.

☒ Life sciences ☐ Behavioural & social sciences ☐ Ecological, evolutionary & environmental sciences

For a reference copy of the document with all sections, see nature.com/documents/nr-reporting-summary-flat.pdf

Life sciences study design

All studies must disclose on these points even when the disclosure is negative.

Sample size	The determination of sample size for genome sequencing is not applied in this study. Sample size for FISH experiments were determined according to the previously published work in the field (Ling et al. 2006), but was also limited by the availability of the material. Bioinformatic analyses were performed with all available data.
Data exclusions	None
Replication	FISH experiments showing that Super_Scaffold_40 did not co-localize with the chr15 BAC were carried out in two independent experiments and co-localization of Super_Scaffold_40 with the chr13 BAC was carried out in five independent experiments. Potential interchromosomal interactions indicated by Hi-C data were validated in three independent FISH experiments for each Y2-Y3, Y2-X1and Y2-WSB1(chr17) pair.
Randomization	Randomization was not performed in this study. Only one cell line was used (established from a single individual) and all available data was collected in all experiments to fulfill the criteria for the sample size. In strata analysis gametologues were grouped according to the pairwise dS and phylogeny. Grouping in interchromosomal interaction analysis was based on sequence chromosome assginment.
Blinding	Our study was not an intervention study and therefore blinding was not required.

Reporting for specific materials, systems and methods

We require information from authors about some types of materials, experimental systems and methods used in many studies. Here, indicate whether each material, system or method listed is relevant to your study. If you are not sure if a list item applies to your research, read the appropriate section before selecting a response.

Materials & experimental systems

n/a	Involved in the study	
☒	☐	Antibodies
☐	☒	Eukaryotic cell lines
☒	☐	Palaeontology and archaeology
☐	☒	Animals and other organisms
☒	☐	Human research participants
☒	☐	Clinical data
☒	☐	Dual use research of concern

Methods

n/a	Involved in the study	
☒	☐	ChIP-seq
☒	☐	Flow cytometry
☒	☐	MRI-based neuroimaging

Eukaryotic cell lines

Policy information about cell lines

Cell line source(s)	Fibroblast primary cell culture derived from the adult male platypus individual number 1 from NSW field expedition in New South Wales, year 2008.

Authentication	None of the cell lines used were authenticated.
Mycoplasma contamination	Cell lines were not tested for mycoplasma contamination.
Commonly misidentified lines (See ICLAC register)	No commonly misidentified lines were used.

Animals and other organisms

Policy information about studies involving animals; ARRIVE guidelines recommended for reporting animal research

Laboratory animals	Study did not involve laboratory animals.
Wild animals	Study did not involve wild animals.
Field-collected samples	Platypus and echidna male individuals where collected during breeding season at the upper Barnard river area (New South Wales) and euthanised with an intraperitoneal injection of pentobarbitone sodium (Nembutal) at a dose of 0.1 mg/g body weight. Tissues where snap frozen in liquid N2 immediately. Emale01 was collected from Melbourne Zoo and provided by San Diego Zoo Global, with an estimated date of birth of 12/31/67.
Ethics oversight	Platypus and echidna samples (Pmale08, Pmale09, Emale12) were collected under the AEC permits S-49-2006, S-032-2008 and S-2011-146 at the Upper Barnard River (New South Wales, Australia) during the breeding season. Emale01 was collected under San Diego Zoo GlobalIACUC approval 18-024, and is vouchered at the San Diego Natural History Museum.

Note that full information on the approval of the study protocol must also be provided in the manuscript.
